# Pregnancy outcomes and risk of placental malaria after artemisinin-based and quinine-based treatment for uncomplicated falciparum malaria in pregnancy: a WorldWide Antimalarial Resistance Network systematic review and individual patient data meta-analysis

**DOI:** 10.1186/s12916-020-01592-z

**Published:** 2020-06-02

**Authors:** Makoto Saito, Rashid Mansoor, Kalynn Kennon, Anupkumar R. Anvikar, Elizabeth A. Ashley, Daniel Chandramohan, Lauren M. Cohee, Umberto D’Alessandro, Blaise Genton, Mary Ellen Gilder, Elizabeth Juma, Linda Kalilani-Phiri, Irene Kuepfer, Miriam K. Laufer, Khin Maung Lwin, Steven R. Meshnick, Dominic Mosha, Atis Muehlenbachs, Victor Mwapasa, Norah Mwebaza, Michael Nambozi, Jean-Louis A. Ndiaye, François Nosten, Myaing Nyunt, Bernhards Ogutu, Sunil Parikh, Moo Kho Paw, Aung Pyae Phyo, Mupawjay Pimanpanarak, Patrice Piola, Marcus J. Rijken, Kanlaya Sriprawat, Harry K. Tagbor, Joel Tarning, Halidou Tinto, Innocent Valéa, Neena Valecha, Nicholas J. White, Jacher Wiladphaingern, Kasia Stepniewska, Rose McGready, Philippe J. Guérin

**Affiliations:** 1 WorldWide Antimalarial Resistance Network (WWARN), Oxford, UK; 2 Infectious Diseases Data Observatory (IDDO), Oxford, UK; 3 Centre for Tropical Medicine and Global Health, Nuffield Department of Medicine, University of Oxford, Oxford, UK; 4 ICMR-National Institute of Malaria Research, New Delhi, India; 5 Lao-Oxford-Mahosot Hospital-Wellcome Trust Research Unit, Vientiane, Lao PDR; 6 London School of Hygiene and Tropical Medicine, London, UK; 7 Center for Vaccine Development and Global Health, University of Maryland School of Medicine, Baltimore, MD USA; 8 Medical Research Council Unit, The Gambia at the London School of Hygiene & Tropical Medicine, Banjul, The Gambia; 9 Department of Epidemiology and Public Health, Swiss Tropical and Public Health Institute, Basel, Switzerland; 10 University of Basel, Basel, Switzerland; 11 University Center of General Medicine and Public Health, Lausanne, Switzerland; 12 Shoklo Malaria Research Unit, Mahidol-Oxford Tropical Medicine Research Unit, Faculty of Tropical Medicine, Mahidol University, Mae Sot, Tak Thailand; 13 Kenya Medical Research Institute, Nairobi, Kenya; 14 Department of Medicine, University of Malawi College of Medicine, Blantyre, Malawi; 15 Department of Epidemiology, Gillings School of Global Public Health, University of North Carolina, Chapel Hill, NC USA; 16 Ifakara Health Institute, Dar es Salaam, Tanzania; 17 Department of Pathology, University of Washington, Seattle, WA USA; 18 Infectious Disease Research Collaboration, Makerere University, Kampala, Uganda; 19 Department of Clinical Sciences, Tropical Diseases Research Centre, Ndola, Zambia; 20 Department of Parasitology, Universite Cheikh Anta Diop, Dakar, Senegal; 21 Duke Global Health Institute, Duke University, Durham, NC USA; 22 Yale School of Public Health, New Haven, CT USA; 23 Myanmar-Oxford Clinical Research Unit, Yangon, Myanmar; 24 Institut Pasteur du Cambodge, Phnom Penh, Cambodia; 25 Department of Obstetrics and Gynecology, Division of Woman and Baby, University Medical Center Utrecht, Utrecht, The Netherlands; 26 School of Medicine, University of Health and Allied Sciences, Ho, Ghana; 27 Mahidol–Oxford Tropical Medicine Research Unit (MORU), Faculty of Tropical Medicine, Mahidol University, Bangkok, Thailand; 28 Clinical Research Unit of Nanoro, Institut de Recherche en Sciences de la Santé, Nanoro, Burkina Faso

**Keywords:** Falciparum malaria, Pregnancy, Treatment, Safety, Stillbirth, Small for gestational age, Preterm birth, Systematic review, Artemisinin, Quinine

## Abstract

**Background:**

Malaria in pregnancy, including asymptomatic infection, has a detrimental impact on foetal development. Individual patient data (IPD) meta-analysis was conducted to compare the association between antimalarial treatments and adverse pregnancy outcomes, including placental malaria, accompanied with the gestational age at diagnosis of uncomplicated falciparum malaria infection.

**Methods:**

A systematic review and one-stage IPD meta-analysis of studies assessing the efficacy of artemisinin-based and quinine-based treatments for patent microscopic uncomplicated falciparum malaria infection (hereinafter uncomplicated falciparum malaria) in pregnancy was conducted. The risks of stillbirth (pregnancy loss at ≥ 28.0 weeks of gestation), moderate to late preterm birth (PTB, live birth between 32.0 and < 37.0 weeks), small for gestational age (SGA, birthweight of < 10th percentile), and placental malaria (defined as deposition of malaria pigment in the placenta with or without parasites) after different treatments of uncomplicated falciparum malaria were assessed by mixed-effects logistic regression, using artemether-lumefantrine, the most used antimalarial, as the reference standard. Registration PROSPERO: CRD42018104013.

**Results:**

Of the 22 eligible studies (*n* = 5015), IPD from16 studies were shared, representing 95.0% (*n* = 4765) of the women enrolled in literature. Malaria treatment in this pooled analysis mostly occurred in the second (68.4%, 3064/4501) or third trimester (31.6%, 1421/4501), with gestational age confirmed by ultrasound in 91.5% (4120/4503). Quinine (*n* = 184) and five commonly used artemisinin-based combination therapies (ACTs) were included: artemether-lumefantrine (*n* = 1087), artesunate-amodiaquine (*n* = 775), artesunate-mefloquine (*n* = 965), and dihydroartemisinin-piperaquine (*n* = 837). The overall pooled proportion of stillbirth was 1.1% (84/4361), PTB 10.0% (619/4131), SGA 32.3% (1007/3707), and placental malaria 80.1% (2543/3035), and there were no significant differences of considered outcomes by ACT. Higher parasitaemia before treatment was associated with a higher risk of SGA (adjusted odds ratio [aOR] 1.14 per 10-fold increase, 95% confidence interval [CI] 1.03 to 1.26, *p* = 0.009) and deposition of malaria pigment in the placenta (aOR 1.67 per 10-fold increase, 95% CI 1.42 to 1.96, *p* < 0.001).

**Conclusions:**

The risks of stillbirth, PTB, SGA, and placental malaria were not different between the commonly used ACTs. The risk of SGA was high among pregnant women infected with falciparum malaria despite treatment with highly effective drugs. Reduction of malaria-associated adverse birth outcomes requires effective prevention in pregnant women.

## Background

Malaria during pregnancy has adverse impacts on the foetus including increased risks of pregnancy loss (i.e. miscarriage or stillbirth) [1, 2], preterm birth (PTB) [3, 4], intrauterine growth restriction (IUGR), and small for gestational age (SGA) [3, 5, 6]. PTB is the major reason for neonatal and infant mortality globally, and the highest burden of mortality occurs in low- and middle-income countries, where malaria is endemic [7]. IUGR leads to a higher risk of stillbirth [8] and is associated with increased short-term mortality and morbidity [7]. SGA is a proxy for IUGR, and risk extends beyond infancy as they are associated with a higher risk of metabolic disorders, and possibly mental disorders or cognitive impairment in later adult life [9, 10].

The most widely reported adverse impact of malaria in pregnancy is low birthweight (LBW), commonly defined as birthweight less than 2500 g and used as a proxy for foetal growth [11, 12]. However, LBW does not distinguish between being “born to early” (or PTB) or “born to small” (or IUGR), or both, which are associated with different neonatal mortality and morbidity. For the purpose of summarizing international evidence, LBW is a poor proxy as newborns in Asia are generally smaller than Africa [13], and gestational age distribution can also vary between different regions [14]. A better proxy for IUGR is SGA, defined as < 10th percentile of the standard growth chart, which takes account of sex and gestational age. An advance in the last decade is that an international standard foetal growth chart defining SGA has become available [15] permitting standardized comparison using SGA to summarize the evidence of the impact of malaria on foetal growth.

Placental malaria (i.e. sequestration of malaria parasites in the placenta) is one of the primary pathogenic mechanisms by which malaria during pregnancy can cause adverse effects to the foetus [16, 17]. Many studies have been conducted to assess the relationship between placental malaria and adverse pregnancy outcomes and have confirmed the association [18–20]. Placental malaria is a direct consequence of malaria infection and is therefore an efficacy outcome measure after treatment or intermittent preventive treatment in pregnancy (IPTp) [21, 22].

Artemisinin-based combination therapies (ACTs) are highly effective with expected ≥ 95% treatment success (clearance of peripheral parasitaemia) in pregnancy [23, 24]. However, few studies have been powered to specifically explore the impact of different treatments on adverse pregnancy outcomes and placental malaria.

In this study, individual patient data (IPD) from treatment efficacy studies have been pooled to describe the risks of adverse pregnancy outcomes (i.e. indirect consequences) and placental malaria (i.e. a direct consequence) after the treatment of uncomplicated falciparum malaria during pregnancy accompanied with reliable gestational age, mostly estimated by ultrasound, at the times of diagnosis of uncomplicated falciparum malaria infection and delivery. This study aims to compare the risks of stillbirth, PTB, SGA, and placental malaria after different treatments for patent microscopic falciparum malaria (uncomplicated or asymptomatic) mainly in the second and third trimester, using artemether-lumefantrine (AL), the most used ACT, as the reference standard.

## Methods

### Search strategy and inclusion criteria

A systematic review and IPD meta-analysis on the efficacy of artemisinin-based and quinine-based treatments on uncomplicated falciparum malaria in pregnancy was conducted with registration to PROSPERO (CRD42018104013). Briefly, a combination of five components were searched in seven databases (Medline, Embase, Global Health, Cochrane Library, Scopus, Web of Science, and Literatura Latino Americana em Ciências da Saúde) and two clinical trial registries (International Clinical Trials Registry Platform and ClinicalTrials.gov) on 26 April 2019 without any restrictions on language or publication year: malaria, pregnancy, treatment or names of antimalarial drugs, study design (interventional or observational cohort studies), and outcome types (efficacy). Studies were included if *Plasmodium falciparum* parasitaemia was confirmed by microscopy before treatment, the length of active follow-up was ≥ 28 days, polymerase chain reaction (PCR) was used to classify recurrence of falciparum malaria, gestational age at detection of parasitaemia and delivery was verified, and pregnant women were followed up until delivery for assessing pregnancy outcomes. Investigators were invited to join this project and share the IPD with the WorldWide Antimalarial Resistance Network (WWARN), which were then standardized for the IPD meta-analysis as described in the published protocol [25].

### Definition of outcomes

Miscarriage was defined as foetal death before 28.0 weeks of gestation, and stillbirth was defined as foetal death before birth resulting in delivery of a newborn with no signs of life at ≥ 28.0 weeks of gestation (regardless of birthweight) [26, 27]. PTB was defined as birth of a live infant before 37.0 weeks of gestation [28]: extremely PTB (< 28.0 weeks), very PTB (≥ 28.0 to < 32.0 weeks), and moderate to late PTB (≥ 32.0 to < 37.0 weeks) [29]. SGA was defined as birthweight lower than the 10th percentile [30, 31] of the INTERGROWTH-21st international standard growth chart [15].

Placental histopathology was categorized according to the malaria parasites and malaria pigment as follows [32]: no infection (both parasites and pigment are negative), acute infection (only parasites are present), past infection (only pigment present), and chronic infection (both parasites and pigment are present). The presence of malaria pigment (i.e. both past and chronic infections) was analysed in order to assess the effect of malaria infection during pregnancy prior to labour as the presence of parasites in the placenta is more closely associated with acute parasitaemia at delivery than parasitaemia earlier in pregnancy [16].

### Inclusion criteria at the individual level

Only singleton births without congenital abnormality were analysed, except for calculating the proportion of congenital abnormality, which included all pregnant women with delivery information. The proportion of miscarriage was calculated excluding malaria episodes at ≥ 28.0 weeks of gestation. PTB included only live singleton births and excluded pregnant women who had the first recorded malaria at ≥ 28.0 weeks of gestation (for extremely PTB), ≥ 32.0 (for very PTB), or ≥ 37.0 (for moderate to late PTB). Only live singleton births delivered < 43.0 weeks of gestation were included for assessing SGA. Newborns whose birthweight was assessed within 3 days were included in the birthweight analyses.

### Statistical analysis

All statistical analyses were performed using R (version 3.2.5, R Foundation for Statistical Computing, Vienna, Austria) or Stata MP 15.1 (StataCorp, College Station, TX, USA). One-stage IPD meta-analysis was conducted: univariable and multivariable mixed-effects logistic regression models were used to model risk of (i) stillbirth, (ii) PTB, (iii) SGA, and (iv) placental malaria. Study site was fitted as a random intercept in these models. Heterogeneity between studies was assessed by intraclass correlation. Multiple imputation (MI) was used for handling missing values of covariates to test the effect of exclusion of patients with missing data on our main conclusions. For the key analysis, both MI (presented as the main model) and complete case analyses are presented. MI was conducted in Stata MP 15.1 with 20 imputations using *mi impute mvn* command to impute missing values of HIV status (~ 18% missing) and height (~ 14% missing). As recommended when MI is used, the Wald test was used for model building [33, 34] by backward elimination using *p* < 0.05 as the cut-off (see MI procedure in Additional file 1). Treatment was always included in the model regardless of its significance using AL, which is the most used ACT, as the reference. Comparisons of each pair of drugs were not conducted to avoid multiple testing. HIV status was always adjusted as a confounder except in analysis of placental malaria [35]. For placental malaria, malaria transmission intensity was included in the multivariable model as an a priori confounder, and interaction between parity and malaria transmission was assessed because pregnancy-specific (parity-dependent) immunity can be different depending on malaria transmission intensity. If the number of overall observations (or outcomes) was small, multivariable analysis was not attempted and only univariable mixed-effects logistic regression (by treatment) or pooled proportions with 95% confidence interval (CI) taking account for study site (pooled by DerSimonian and Laird’s random effects after Freeman-Tukey double arcsine transformation) were presented [36]. Raw numbers were presented for references, but these fractions were not necessarily the same as the shown proportions, which were pooled by random effects. Risk of bias assessment is available in Additional file 2. Two post hoc sensitivity analyses (one regarding gestational age and the other stratified by geographical region) were conducted (Additional file 3).

As the majority of the studies included second (≥ 14.0 to < 28.0 weeks) or third trimester (≥ 28.0 weeks) women, the gestational period between ≥ 14.0 and < 37.0 weeks was divided into four periods that had a similar number of women, and women included for a malaria episode before 14.0 weeks and after 37.0 weeks constituted a fifth and a sixth group, respectively. Gravidity (G) and previous history of pregnancy loss were jointly categorized (G1, G2 with no loss, G ≥ 3 with no loss, G2 with 1 loss, G ≥ 3 with 1 loss, and G ≥ 3 with ≥ 2 losses) and always adjusted in the multivariable analyses as a confounder for assessing stillbirth, PTB, and SGA [8, 35]. Previous history of pregnancy loss was estimated by (enrolement gravidity – parity − 1), with a lower limit of 0. Age was categorized as < 20, 20–24, 25–29, 30–34, and ≥ 35 years [37].

## Results

### Study inclusion

**Fig. 1:**
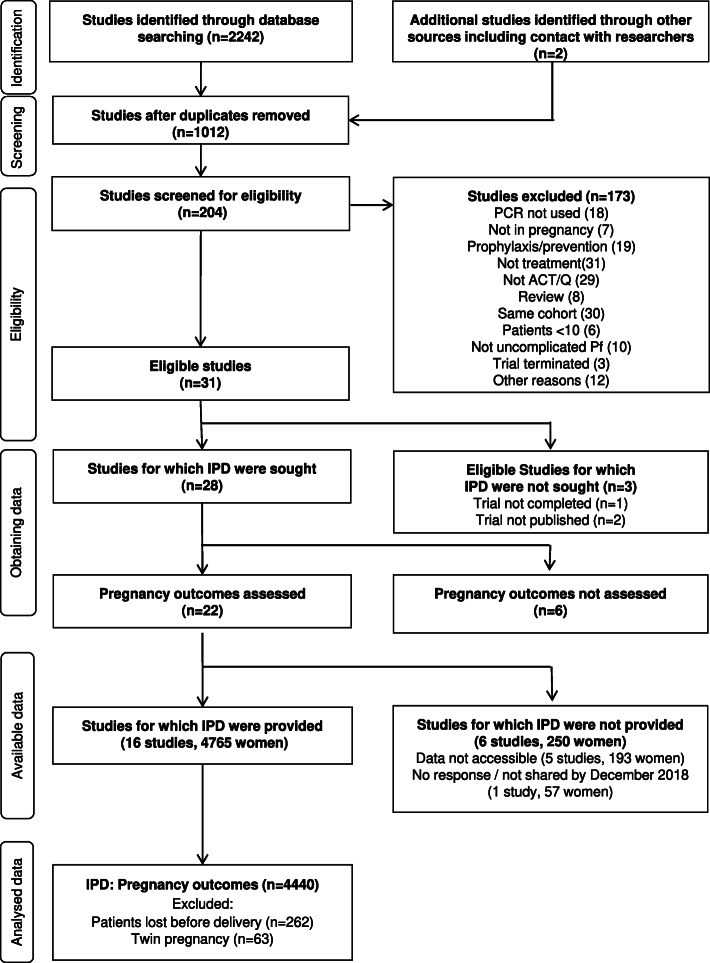
Flowchart of eligible studies included in the analysis

Of the 28 identified studies in the literature with PCR-corrected efficacy, 22 studies assessed pregnancy outcomes, resulting in a total of 5015 women enrolled in those trials. Data of 4765 women from 16 studies, representing 95.0% of the total target, were shared to the WWARN repository and pooled for this IPD meta-analysis. Delivery information was available for 4503 women (3607 in Africa and 896 in Asia) and missing for 5.5% (262/4765) including 256 lost to follow-up and six maternal deaths. Of the 4501 women with estimated gestational age, 68.1% (3064/4501) had documented falciparum malaria in the second trimester and 31.6% (1421/4501) in the third trimester. Only 16 malaria episodes (0.3%, 16/4501) were reported in the first trimester. After excluding twin pregnancies (1.4%, 63/4503), 4440 women with singleton deliveries were included (Fig. 1).

Ultrasound was used to estimate gestational age in eight studies (Additional Table 1), comprising 91.5% (4120/4503) of the women. One study did not have information on gestational age. Another study assessed EGA by ultrasound but reported EGA only in weeks (details are available in Additional file 3).

Histopathology of the placenta was assessed in 3128 pregnant women in six studies with PCR-corrected efficacy in the literature, and IPD of 3033 women (97.0%) in four studies were shared and analysed.

### Congenital abnormality

The overall proportion of congenital abnormality pooled by DerSimonian and Laird’s random effects was 0.4% (58/4440, 95% CI 0.1 to 0.9, *I*^2^ 39%). However, congenital abnormalities were only reported in live born infants (*n* = 4335). As they are a known contributor to stillbirths, congenital abnormalities were likely to be underestimated. There was no difference among different treatments (*p* = 0.68). No further analyses were conducted because of the small number of cases. The 58 pregnancies with congenital abnormality were excluded from the analyses that follow.

### Miscarriage

The overall pooled proportion of miscarriage was 0.0% (21/2932, 95% CI 0.0 to 0.1, *I*^2^ 0%, Additional Figure 1) among 2932 pregnant women who had malaria < 28.0 weeks of gestation (mean 21.4, standard deviation 3.5 weeks) and were followed up to the end of pregnancy. There was no difference among different treatments (*p* = 0.60). No further analyses were conducted because of the small number of cases.

### Stillbirth

The overall pooled proportion of stillbirth was 1.1% (84/4361, 95% CI 0.6 to 1.7, *I*^2^ 29%, Additional Figure 2), among pregnant women who were followed up until delivery, excluding those who had a miscarriage. Pregnant women for whom there was no information on EGA (*n* = 27) were excluded from the subsequent analysis on stillbirth.

Of the 4334 women, nine antimalarial treatments were used and included in the analyses on stillbirth: AL (*n* = 1087), artesunate-amodiaquine (ASAQ, *n* = 775), artesunate-mefloquine (ASMQ, *n* = 965), dihydroartemisinin-piperaquine (DP, *n* = 837), artesunate-sulfadoxine-pyrimethamine (ASSP, *n* = 154), artesunate monotherapy (AS, *n* = 193), artesunate-atovaquone-proguanil (AAP, *n* = 81), quinine monotherapy (*n* = 184), and quinine with clindamycin (QC, *n* = 58) (Table 1).

**Table 1:** Baseline characteristics of pregnant women assessed for stillbirth

Characteristic	All		AL		ASAQ		ASMQ		DP		AAP		AS		ASSP		Q		QC	
	*N*	% (*N*) Mean (SD)	*N*	% (*N*) Mean (SD)	*N*	% (*N*) Mean (SD)	*N*	% (*N*) Mean (SD)	*N*	% (*N*) Mean (SD)	*N*	% (*N*) Mean (SD)	*N*	% (*N*) Mean (SD)	*N*	% (*N*) Mean (SD)	*N*	% (*N*) Mean (SD)	*N*	% (*N*) Mean (SD)
EGA (week)	4334	24.6 (5.7)	1087	24.3 (5.7)	775	24.1 (5.1)	965	25.3 (5.3)	837	24.4 (5.7)	81	26.2 (7.1)	193	26.6 (7.4)	154	24.4 (5.8)	184	23.8 (6.3)	58	26.8 (6.5)
Age group <20	4334	32.0 (1388)	1087	36.5 (397)	775	31.7 (246)	965	28.1 (271)	837	39.8 (333)	81	28.4 (23)	193	19.2 (37)	154	20.1 (31)	184	20.7 (38)	58	20.7 (12)
20–24		32.3 (1399)		31.1 (338)		31.6 (245)		33.6 (324)		30.0 (251)		27.2 (22)		24.9 (48)		55.2 (85)		39.7 (73)		22.4 (13)
25–29		19.3 (836)		17.8 (194)		20.1 (156)		21.2 (205)		16.6 (139)		22.2 (18)		21.2 (41)		17.5 (27)		20.1 (37)		32.8 (19)
30–34		9.7 (419)		8.1 (88)		10.7 (83)		10.6 (102)		8.4 (70)		13.6 (11)		14.5 (28)		3.9 (6)		13.0 (24)		12.1 (7)
>35 (years)		6.7 (292)		6.4 (70)		5.8 (45)		6.5 (63)		5.3 (44)		8.6 (7)		20.2 (39)		3.2 (5)		6.5 (12)		12.1 (7)
Gravidity 1	4297	35.4 (1519)	1053	35.1 (370)	774	36.4 (282)	965	33.2 (320)	837	38.4 (321)	81	29.6 (24)	193	27.5 (53)	154	48.7 (75)	182	32.4 (59)	58	25.9 (15)
2		23.4 (1005)		23.2 (244)		22.5 (174)		24.1 (233)		24.9 (208)		21.0 (17)		15.0 (29)		24.7 (38)		25.8 (47)		25.9 (15)
≥3		41.3 (1773)		41.7 (439)		41.1 (318)		42.7 (412)		36.8 (308)		49.4 (40)		57.5 (111)		26.6 (41)		41.8 (76)		48.3 (28)
Parity 0	4329	42.7 (1847)	1083	42.6 (461)	774	41.1 (318)	965	42.6 (411)	837	47.6 (398)	81	33.3 (27)	193	30.1 (58)	154	56.5 (87)	184	37.0 (68)	58	32.8 (19)
1		21.8 (945)		20.9 (226)		22.4 (173)		20.9 (202)		22.6 (189)		24.7 (20)		19.7 (38)		23.4 (36)		25.0 (46)		25.9 (15)
≥2		35.5 (1537)		36.6 (396)		36.6 (283)		36.5 (352)		29.9 (250)		42.0 (34)		50.3 (97)		20.1 (31)		38.0 (70)		41.4 (24)
Height (cm)	3809	155.8 (6.9)	934	156.1 (6.6)	775	157.9 (6.5)	927	156.1 (7.3)	837	155.3 (6.3)	33	150.2 (8.3)	136	151.7 (5.6)	118	150.0 (7.0)	49	153.3 (5.1)	0	
Weight (kg)	4333	53.9 (8.3)	1086	53.7 (7.4)	775	56.3 (8.8)	965	54.0 (8.5)	837	54.4 (8.2)	81	49.8 (6.6)	193	49.5 (6.4)	154	47.8 (6.9)	184	55.2 (9.4)	58	49.8 (5.5)
BMI <18.5	3809	6.3 (239)	934	5.8 (54)	775	4.9 (38)	927	6.8 (63)	837	5.5 (46)	33	6.1 (2)	136	8.1 (11)	118	18.6 (22)	49	6.1 (3)	0	
18.5–24.9		79.9 (3044)		84.7 (791)		78.8 (611)		77.9 (722)		77.8 (651)		78.8 (26)		83.8 (114)		72.0 (85)		89.8 (44)		
25.0–29.9		12.0 (458)		8.9 (83)		12.4 (96)		13.4 (124)		15.4 (129)		9.1 (3)		7.4 (10)		9.3 (11)		4.1 (2)		
≥30 (kg/m^2^)		1.8 (68)		0.6 (6)		3.9 (30)		1.9 (18)		1.3 (11)		6.1 (2)		0.7 (1)		0.0 (0)		0.0 (0)		
Fever (temperature >37.5°C)	4292	9.2 (395)	1086	8.2 (89)	775	4.8 (37)	964	9.8 (94)	834	4.2 (35)	81	24.7 (20)	193	25.4 (49)	117	24.8 (29)	184	16.8 (31)	58	19.0 (11)
Haemoglobin on day 0 (g/dL)	4308	10.0 (1.4)	1075	10.2 (1.4)	775	10.0 (1.3)	963	9.9 (1.4)	834	10.0 (1.4)	80	9.4 (1.5)	193	9.5 (1.5)	152	9.3 (1.4)	179	10.2 (1.8)	57	9.1 (1.6)
Parasitaemia (log_10_/μL)	4334	3.0 (0.9)	1087	3.1 (0.8)	775	2.8 (0.8)	965	3.0 (0.8)	837	2.9 (0.8)	81	3.6 (1.1)	193	3.3 (1.0)	154	3.4 (0.8)	184	3.4 (0.9)	58	3.3 (1.1)
Presence of gametocytes	4271	3.8 (161)	1083	3.9 (42)	775	3.0 (23)	946	1.7 (16)	837	3.8 (32)	81	3.7 (3)	189	10.1 (19)	118	3.4 (4)	184	9.2 (17)	58	8.6 (5)
Mixed infection	4334	0.7 (32)	1087	0.9 (10)	775	0.0 (0)	965	0.0 (0)	837	0.7 (6)	81	1.2 (1)	193	6.2 (12)	154	0.0 (0)	184	0.0 (0)	58	5.2 (3)
HIV infection	2730	1.3 (35)	808	1.4 (11)	573	0.5 (3)	570	0.2 (1)	668	0.9 (6)	0		0		27	22.2 (6)	84	9.5 (8)	0	

*AAP* artesunate with atovaquone-proguanil, *AL* artemether-lumefantrine, *AS* artesunate monotherapy, *ASAQ* artesunate-amodiaquine, *ASMQ* artesunate-mefloquine, *ASSP* artesunate-sulfadoxine-pyrimethamine, *BMI* body mass index, *DP* dihydroartemisinin-piperaquine, *EGA* estimated gestational age, *HIV* human immunodeficiency virus, *Q* quinine monotherapy, *QC* quinine with clindamycin, *SD* standard deviation

The median age was 22 (interquartile range 19–27), and gravidity was 2 (interquartile range 1–4). Malaria episodes in the included studies were mostly in the second (68.0%, 2949/4334) or third trimester (31.6%, 1371/4334). The majority of the episodes (99.3%, 4302/4334) were *P. falciparum* mono-infections (Table 1). A small proportion (35/3019 assessed) were HIV positive, and these women had not received antiretroviral treatment during pregnancy. Only eight women received cotrimoxazole prophylaxis. HIV results were unavailable for 643 women enrolled in nine studies conducted on the Thailand-Myanmar border, where the seroprevalence of HIV is < 0.5% [38]. They were thus regarded as HIV negative in the following analyses.

In both the univariable and multivariable analyses, different antimalarial treatments were not associated with different risk of stillbirth using AL as the reference (Table 2). In the multivariable analysis adjusted for HIV status and gravidity combined with a previous history of pregnancy loss, the risk of stillbirth was higher in women with HIV infection (adjusted odds ratio [aOR] 4.35, 95% CI 1.15 to 16.52, *p* = 0.03) and lower in multigravid women who had no previous pregnancy loss (aOR 0.51, 95% CI 0.27 to 0.97, *p* = 0.04) compared with primigravidae. Higher haemoglobin on day 0 of the malaria episode was associated with a higher risk of stillbirth in the multiple imputation model, but not in the univariable and complete case multivariable model.

**Table 2:** Univariable and multivariable logistic regression on the risk of stillbirth

Baseline characteristic	Proportion (%)	Univariable		Multivariable (complete case)		Multivariable (MI)	
		OR (95% CI)	*p* value	aOR (95% CI)	*p* value	aOR (95% CI)	*p* value
Treatment							
AL	19/1087 (2%)	Reference		Reference		Reference	
AAP	1/81 (1%)	0.61 (0.08–4.93)	0.64	0.99 (0.13–7.67)	0.99	0.82 (0.10–6.46)	0.85
AS	2/193 (1%)	0.56 (0.12–2.57)	0.45	0.80 (0.18–3.61)	0.77	0.67 (0.15–3.02)	0.60
ASAQ	13/775 (2%)	0.89 (0.42–1.88)	0.76	0.96 (0.40–2.32)	0.93	1.00 (0.47–2.10)	0.99
ASMQ	23/965 (2%)	1.20 (0.62–2.32)	0.58	1.58 (0.76–3.25)	0.22	1.38 (0.70–2.70)	0.35
ASSP	4/154 (3%)	1.43 (0.42–4.81)	0.57	1.23 (0.34–4.49)	0.75	1.35 (0.42–4.41)	0.62
DP	19/837 (2%)	1.09 (0.54–2.18)	0.81	1.36 (0.65–2.85)	0.41	1.22 (0.60–2.47)	0.58
Q	2/184 (1%)	0.51 (0.11–2.34)	0.39	0.71 (0.16–3.25)	0.66	0.51 (0.11–2.33)	0.39
QC	1/58 (2%)	0.81 (0.09–7.15)	0.85	1.40 (0.18–11.01)	0.75	1.12 (0.14–9.24)	0.92
EGA at malaria episode							
4.0–13.9	0/38 (0%)	No data					
14.0–19.9	22/896 (2%)	Reference					
20.0–23.9	20/1065 (2%)	0.72 (0.39–1.33)	0.30				
24.0–27.9	21/964 (2%)	0.82 (0.45–1.51)	0.53				
28.0–36.9	19/1319 (1%)	0.51 (0.27–0.95)	0.04				
≥ 37.0 (weeks)	2/51 (4%)	1.63 (0.37–7.32)	0.52				
Age group							
< 20	27/1388 (2%)	Reference					
20–25	27/1399 (2%)	0.97 (0.56–1.66)	0.91				
25–30	18/836 (2%)	1.10 (0.60–2.02)	0.76				
30–35	6/419 (1%)	0.73 (0.30–1.77)	0.48				
≥ 35 (years)	6/292 (2%)	1.03 (0.42–2.54)	0.95				
Pregnancy history							
G1	33/1519 (2%)	Reference		Reference		Reference	
G2 with no loss	11/741 (1%)	0.68 (0.34–1.35)	0.27	0.52 (0.23–1.15)	0.11	0.63 (0.31–1.26)	0.19
G ≥ 3 with no loss	15/1165 (1%)	0.60 (0.33–1.11)	0.11	0.47 (0.23–0.98)	0.04	0.51 (0.27–0.97)	0.04
G2 with 1 loss	8/264 (3%)	1.28 (0.57–2.84)	0.55	1.12 (0.46–2.74)	0.81	1.35 (0.60–3.01)	0.47
G ≥ 3 with 1 loss	11/462 (2%)	1.05 (0.53–2.11)	0.89	0.60 (0.25–1.49)	0.27	0.95 (0.46–1.93)	0.88
G ≥ 3 with ≥ 2 losses	5/145 (3%)	1.45 (0.55–3.81)	0.45	0.98 (0.28–3.37)	0.97	1.33 (0.49–3.64)	0.58
Weight (kg)	84/4334 (2%)	1.01 (0.99–1.04)	0.34				
Height (cm)	73/3809 (2%)	0.99 (0.95–1.02)	0.42				
BMI (kg/m^2^)							
< 18.5	8/239 (3%)	2.16 (1.01–4.62)	0.048				
18.5–24.9	49/3044 (2%)	Reference					
25.0–29.9	12/458 (3%)	1.40 (0.71–2.77)	0.33				
≥ 30.0	4/68 (6%)	2.86 (0.93–8.80)	0.07				
HIV infection							
Yes	3/35 (9%)	5.41 (1.57–18.65)	0.008	6.06 (1.69–21.68)	0.006	4.35 (1.15–16.52)	0.03
No	61/3627 (2%)	Reference		Reference		Reference	
Parasitaemia (log_10_/μL)	84/4334 (2%)	1.16 (0.90–1.51)	0.26				
Fever > 37.5 °C							
Yes	8/395 (2%)	1.20 (0.56–2.56)	0.64				
No	73/3897 (2%)	Reference					
Haemoglobin (g/dL)	84/4334 (2%)	1.13 (0.97–1.32)	0.12	1.14 (0.94–1.37)	0.18	1.20 (1.02–1.41)	0.03
Gametocytaemia							
Yes	2/161 (1%)	0.67 (0.17–2.75)	0.58				
No	79/4110 (2%)	Reference					
Mixed infection							
Yes	0/32 (0%)	No data					
No	84/4302 (2%)	Reference					
Malaria transmission							
Low	12/993 (1%)	0.44 (0.21–0.90)	0.03				
Moderate	57/2348 (2%)	Reference					
High	15/993 (2%)	0.61 (0.30–1.26)	0.18				

Intraclass correlation, < 0.01. *AAP* artesunate with atovaquone-proguanil, *AL* artemether-lumefantrine, *aOR* adjusted odds ratio, *AS* artesunate monotherapy, *ASAQ* artesunate-amodiaquine, *ASMQ* artesunate-mefloquine, *ASSP* artesunate-sulfadoxine-pyrimethamine, *BMI* body mass index, *CI* confidence interval, *DP* dihydroartemisinin-piperaquine, *EGA* estimated gestational age, *G* gravidity, *HIV* human immunodeficiency virus, *MI* multiple imputation, *OR* odds ratio, *Q* quinine monotherapy, *QC* quinine with clindamycin

### Preterm birth

The overall pooled proportions of extremely PTB (< 28.0 weeks), very PTB (≥ 28.0 to < 32.0 weeks), and moderate to late PTB (≥ 32.0 to < 37.0 weeks) were 0.0% (8/2488, 95% CI 0.0 to 0.0, *I*^2^ 0%), 0.4% (47/3131, 95% CI 0.1 to 0.9, *I*^2^ 11%), and 10.0% (619/4131, 95% CI 7.0 to 13.4, *I*^2^ 88%, Additional Figure 3), respectively. Since the number of outcomes of extremely PTB and very PTB was small even when pooled, they were not analysed further. The risk of moderate to late PTB among different treatments used in five different pregnancy periods was investigated.

The same nine antimalarial treatments were included in this analysis: AL (*n* = 1035), ASAQ (*n* = 747), ASMQ (*n* = 926), DP (*n* = 804), ASSP (*n* = 147), AS (*n* = 174), AAP (*n* = 76), quinine (*n* = 171), and QC (*n* = 51). Baseline characteristics (Additional Table 2) were similar to that of pregnant women included in the analysis of stillbirth. The gestational period of pregnancy at the time of malaria episode was divided into < 14.0 weeks (*n* = 38), 14.0–19.9 weeks (*n* = 863), 20.0–23.9 weeks (*n* = 1023), 24.0–27.9 weeks (*n* = 917), and 28.0–36.9 weeks (*n* = 1290).

In both the univariable and multivariable analyses, different antimalarial treatments were not associated with moderate to late PTB in comparison with AL (Table 3). Malaria between 14.0 and < 20.0 weeks was associated with the lowest risk of PTB and was used as the reference. Malaria in the first trimester was associated with a higher risk of PTB (aOR 3.92, 95% CI 1.38 to 11.13, *p* = 0.01). For each Isuccessive gestational period between 14.0 and < 37.0 weeks, the risk of PTB increased. Taller maternal height (aOR 0.98 per cm, 95% CI 0.96 to 0.99, *p* = 0.01) and higher maternal BMI (aOR 0.94 per kg/m^2^, 95% CI 0.90 to 0.97, *p* < 0.001) were linearly associated with a lower risk of PTB. Teenagers and primigravidae were independently associated with a higher risk of PTB. Compared with primigravidae, the risk of PTB was lower in G2 women with no previous pregnancy loss (aOR 0.65, 95% CI 0.48 to 0.89, *p* = 0.007) and G ≥ 3 women with no loss (aOR 0.67, 95% CI 0.47 to 0.96, *p* = 0.03) or one loss (aOR 0.66, 95% CI 0.44 to 0.99, *p* = 0.05). Compared with women < 20 years old, the risk of PTB was lower in 20–24 year olds (aOR 0.67, 95% CI 0.51 to 0.87, *p* = 0.003), but not different in 25 and older. Compared with moderate malaria transmission areas, the risk of PTB was lower in low transmission areas but not different in high transmission areas. When only studies in sub-Saharan Africa were included (sensitivity analysis), the risk of PTB was higher after ASSP than AL (aOR 4.90, 95% CI 1.23 to 19.48, *p* = 0.02) (Additional file 3).

**Table 3:** Univariable and multivariable logistic regression on the risk of moderate-to-late preterm birth

Baseline characteristic	Proportion (%)	Univariable		Multivariable (complete case)		Multivariable (MI)	
		OR (95% CI)	*p* value	aOR (95% CI)	*p* value	aOR (95% CI)	*p* value
Treatment							
AL	156/1035 (15%)	Reference		Reference		Reference	
AAP	4/76 (5%)	0.51 (0.15–1.70)	0.27	2.72 (0.50–14.75)	0.25	0.69 (0.19–2.44)	0.56
AS	15/174 (9%)	1.41 (0.63–3.18)	0.40	2.01 (0.74–5.47)	0.17	1.49 (0.64–3.50)	0.36
ASAQ	124/747 (17%)	1.04 (0.79–1.38)	0.76	1.03 (0.75–1.41)	0.88	1.05 (0.79–1.39)	0.75
ASMQ	128/926 (14%)	1.10 (0.84–1.46)	0.48	0.94 (0.68–1.30)	0.71	1.07 (0.80–1.41)	0.65
ASSP	22/147 (15%)	1.78 (0.87–3.63)	0.11	1.24 (0.50–3.09)	0.64	1.83 (0.88–3.81)	0.10
DP	151/804 (19%)	1.28 (0.98–1.66)	0.07	1.23 (0.93–1.64)	0.15	1.22 (0.93–1.60)	0.15
Q	15/171 (9%)	1.17 (0.57–2.40)	0.68	3.86 (0.92–16.19)	0.07	1.67 (0.79–3.54)	0.18
QC	4/51 (8%)	1.03 (0.26–4.08)	0.96	No data		1.39 (0.34–5.67)	0.65
EGA at malaria episode							
4.0–13.9	5/38 (13%)	3.92 (1.42–10.87)	0.009	2.67 (0.73–9.80)	0.14	3.92 (1.38–11.13)	0.01
14.0–19.9	78/863 (9%)	Reference		Reference		Reference	
20.0–23.9	136/1023 (13%)	1.69 (1.25–2.28)	< 0.001	1.63 (1.17–2.28)	0.004	1.71 (1.25–2.32)	< 0.001
24.0–27.9	142/917 (15%)	2.17 (1.60–2.94)	< 0.001	2.30 (1.64–3.24)	< 0.001	2.37 (1.74–3.23)	< 0.001
28.0–36.9 (weeks)	258/1290 (20%)	3.63 (2.72–4.85)	< 0.001	4.49 (3.23–6.25)	< 0.001	4.33 (3.21–5.84)	< 0.001
Age group							
< 20	284/1323 (21%)	Reference		Reference		Reference	
20–25	154/1330 (12%)	0.55 (0.44–0.68)	< 0.001	0.69 (0.51–0.93)	0.02	0.67 (0.51–0.87)	0.003
25–30	100/803 (12%)	0.60 (0.47–0.78)	< 0.001	1.01 (0.67–1.54)	0.96	0.82 (0.56–1.18)	0.29
30–35	46/401 (11%)	0.54 (0.38–0.76)	< 0.001	0.78 (0.47–1.31)	0.34	0.72 (0.45–1.12)	0.15
≥ 35 (years)	35/274 (13%)	0.75 (0.51–1.11)	0.15	0.87 (0.47–1.62)	0.67	0.99 (0.60–1.64)	0.98
Pregnancy history							
G1	284/1439 (20%)	Reference		Reference		Reference	
G2 with no loss	81/704 (12%)	0.56 (0.43–0.73)	< 0.001	0.68 (0.48–0.96)	0.03	0.65 (0.48–0.89)	0.007
G ≥ 3 with no loss	141/1128 (12%)	0.60 (0.48–0.75)	< 0.001	0.59 (0.40–0.89)	0.01	0.67 (0.47–0.96)	0.03
G2 with 1 loss	41/251 (16%)	0.85 (0.59–1.24)	0.40	1.12 (0.75–1.68)	0.58	0.95 (0.65–1.40)	0.81
G ≥ 3 with 1 loss	51/435 (12%)	0.60 (0.43–0.83)	0.002	0.69 (0.44–1.08)	0.10	0.66 (0.44–0.99)	0.046
G ≥ 3 with ≥ 2 losses	18/137 (13%)	0.94 (0.55–1.58)	0.80	1.20 (0.62–2.32)	0.58	1.00 (0.56–1.79)	1.00
Weight (kg)	618/4130 (15%)	0.98 (0.97–0.99)	0.004				
Height (cm)	579/3655 (16%)	0.99 (0.97–1.00)	0.05	0.98 (0.96–1.00)	0.01	0.98 (0.96–0.99)	0.01
BMI (kg/m^2^)	579/3655 (16%)	0.96 (0.93–1.00)	0.03	0.95 (0.91–0.99)	0.01	0.94 (0.90–0.97)	< 0.001
HIV infection							
Yes	4/31 (13%)	1.57 (0.51–4.73)	0.44	1.88 (0.35–10.15)	0.46	1.22 (0.36–4.09)	0.75
No	527/3462 (15%)	Reference		Reference		Reference	
Parasitaemia (log_10_/μL)	619/4131 (15%)	1.11 (0.99–1.24)	0.07				
Fever > 37.5 °C							
Yes	45/375 (12%)	1.09 (0.77–1.54)	0.62				
No	563/3719 (15%)	Reference					
Haemoglobin (g/dL)	616/4107 (15%)	0.94 (0.88–1.00)	0.04				
Gametocytaemia							
Yes	24/153 (16%)	1.46 (0.92–2.32)	0.11				
No	584/3922 (15%)	Reference					
Mixed infection							
Yes	2/29 (7%)	1.04 (0.24–4.52)	0.96				
No	617/4102 (15%)	Reference					
Malaria transmission							
Low	72/929 (8%)	0.55 (0.33–0.94)	0.03	0.24 (0.10–0.56)	0.001	0.42 (0.22–0.81)	0.009
Moderate	397/2236 (18%)	Reference		Reference		Reference	
High	150/966 (16%)	0.93 (0.54–1.62)	0.80	0.56 (0.28–1.09)	0.09	0.86 (0.47–1.58)	0.63

Intraclass correlation, 0.07. *AAP* artesunate with atovaquone-proguanil, *AL* artemether-lumefantrine, *aOR* adjusted odds ratio, *AS* artesunate monotherapy, *ASAQ* artesunate-amodiaquine, *ASMQ* artesunate-mefloquine, *ASSP* artesunate-sulfadoxine-pyrimethamine, *BMI* body mass index, *CI* confidence interval, *DP* dihydroartemisinin-piperaquine, *EGA* estimated gestational age, *G* gravidity, *HIV* human immunodeficiency virus, *MI* multiple imputation, *OR* odds ratio, *Q* quinine monotherapy, *QC* quinine with clindamycin

### Small for gestational age

Among 4277 live singleton births without congenital abnormality, 3707 births were considered in the analysis after excluding 570 births for which SGA was not assessed (248 without birthweight, 253 birthweight measured > 3 days after delivery, one without information on sex of the baby, 41 born after 43.0 weeks, 27 without EGA at delivery). The overall pooled proportion of SGA was 32.3% (1007/3707, 95% CI 26.7 to 38.2, *I*^2^ 90%, Additional Figure 4).

Antimalarial treatments included were AL (*n* = 973), ASAQ (*n* = 700), ASMQ (*n* = 820), DP (*n* = 716), ASSP (*n* = 129), AS (*n* = 154), AAP (*n* = 55), quinine (*n* = 119), and QC (*n* = 41). Baseline characteristics (Additional Table 3) were similar to pregnant women who were included in the previous analysis of stillbirth and PTB.

In both univariable and multivariable analyses, different antimalarial treatments were not statistically associated with SGA when compared with AL (Table 4). For each successive gestational period between 14.0 and < 37.0 weeks, the risk of SGA decreased, with a significantly lower risk of SGA in women with malaria treated between 28.0 and < 37.0 weeks of gestation. Taller maternal height (aOR 0.95 per cm, 95% CI 0.93 to 0.96, *p* < 0.001) and higher maternal BMI (aOR 0.92 per kg/m^2^, 95% CI 0.89 to 0.95, *p* < 0.001) were associated with a lower risk of SGA. Higher baseline parasitaemia (aOR 1.14 per 10-fold increase, 95% CI 1.03 to 1.26, *p* = 0.009) and co-infection of other malaria species (aOR 2.54, 95% CI 1.07 to 5.99, *p* = 0.03) were associated with a higher risk of SGA. Compared with primigravidae, the risk of SGA was lower in G2 women with no previous pregnancy loss (aOR 0.62, 95% CI 0.49 to 0.78, *p* < 0.001) and G ≥ 3 women with no loss (aOR 0.48, 95% CI 0.39 to 0.60, *p* < 0.001) or one loss (aOR 0.54, 95% CI 0.41 to 0.72, *p* < 0.001).

**Table 4:** Univariable and multivariable logistic regression on the risk of small for gestational age

Baseline characteristic	Proportion (%)	Univariable		Multivariable (complete case)		Multivariable (MI)	
		OR (95% CI)	*p* value	aOR (95% CI)	*p* value	aOR (95% CI)	*p* value
Treatment							
AL	241/973 (25%)	Reference		Reference		Reference	
AAP	25/55 (45%)	1.99 (0.89–4.45)	0.09	0.69 (0.18–2.63)	0.58	1.85 (0.72–4.75)	0.20
AS	56/154 (36%)	1.38 (0.83–2.29)	0.22	1.30 (0.73–2.30)	0.38	1.26 (0.74–2.16)	0.40
ASAQ	164/700 (23%)	1.02 (0.79–1.32)	0.85	0.97 (0.71–1.32)	0.85	1.05 (0.80–1.37)	0.73
ASMQ	257/820 (31%)	0.99 (0.78–1.26)	0.96	1.01 (0.76–1.34)	0.95	1.00 (0.78–1.28)	0.99
ASSP	66/129 (51%)	1.44 (0.85–2.44)	0.17	1.74 (0.94–3.20)	0.08	1.37 (0.79–2.36)	0.27
DP	163/716 (23%)	0.86 (0.67–1.12)	0.26	0.91 (0.68–1.21)	0.51	0.87 (0.67–1.14)	0.31
Q	20/119 (17%)	0.56 (0.31–1.04)	0.07	0.22 (0.05–0.92)	0.04	0.69 (0.32–1.52)	0.36
QC	15/41 (37%)	1.28 (0.51–3.18)	0.60	No data		1.11 (0.37–3.35)	0.86
EGA at malaria episode							
4.0–13.9	10/31 (32%)	0.70 (0.31–1.59)	0.39	0.74 (0.30–1.83)	0.51	0.62 (0.27–1.45)	0.27
14.0–19.9	227/765 (30%)	Reference		Reference		Reference	
20.0–23.9	246/914 (27%)	0.81 (0.65–1.01)	0.06	0.82 (0.63–1.06)	0.14	0.80 (0.63–1.00)	0.05
24.0–27.9	219/820 (27%)	0.74 (0.59–0.94)	0.01	0.83 (0.63–1.10)	0.20	0.80 (0.63–1.01)	0.07
28.0–36.9	289/1138 (25%)	0.60 (0.48–0.75)	< 0.001	0.69 (0.53–0.90)	0.006	0.70 (0.55–0.87)	0.002
≥ 37.0 (weeks)	16/39 (41%)	1.13 (0.56–2.27)	0.74	0.59 (0.15–2.23)	0.43	1.36 (0.66–2.84)	0.41
Age group							
< 20	379/1202 (32%)	Reference					
20–25	332/1185 (28%)	0.65 (0.54–0.79)	< 0.001				
25–30	170/717 (24%)	0.52 (0.42–0.66)	< 0.001				
30–35	63/357 (18%)	0.37 (0.27–0.50)	< 0.001				
≥ 35 (years)	63/246 (26%)	0.55 (0.40–0.77)	< 0.001				
Pregnancy history							
G1	444/1316 (34%)	Reference		Reference		Reference	
G2 with no loss	154/614 (25%)	0.58 (0.47–0.73)	< 0.001	0.63 (0.49–0.83)	< 0.001	0.62 (0.49–0.78)	< 0.001
G ≥ 3 with no loss	186/1011 (18%)	0.40 (0.33–0.50)	< 0.001	0.48 (0.37–0.63)	< 0.001	0.48 (0.39–0.60)	< 0.001
G2 with 1 loss	76/225 (34%)	0.81 (0.59–1.11)	0.19	0.75 (0.53–1.07)	0.12	0.82 (0.59–1.12)	0.21
G ≥ 3 with 1 loss	88/387 (23%)	0.46 (0.35–0.61)	< 0.001	0.57 (0.42–0.79)	< 0.001	0.54 (0.41–0.72)	< 0.001
G ≥ 3 with ≥ 2 losses	40/117 (34%)	0.76 (0.50–1.15)	0.19	0.79 (0.47–1.32)	0.37	0.79 (0.51–1.21)	0.27
Weight (kg)	1007/3707 (27%)	0.95 (0.94–0.96)	< 0.001				
Height (cm)	891/3347 (27%)	0.95 (0.94–0.96)	< 0.001	0.95 (0.93–0.96)	< 0.001	0.95 (0.93–0.96)	< 0.001
BMI (kg/m^2^)	891/3347 (27%)	0.92 (0.89–0.95)	< 0.001	0.92 (0.89–0.96)	< 0.001	0.92 (0.89–0.95)	< 0.001
HIV infection							
Yes	6/24 (25%)	0.92 (0.35–2.42)	0.87	2.45 (0.62–9.73)	0.20	1.04 (0.43–2.55)	0.92
No	849/3105 (27%)	Reference		Reference		Reference	
Parasitaemia (log_10_/μL)	1007/3707 (27%)	1.24 (1.13–1.36)	< 0.001	1.20 (1.07–1.35)	0.002	1.14 (1.03–1.26)	0.009
Fever > 37.5 °C							
Yes	112/325 (34%)	1.14 (0.88–1.47)	0.33				
No	888/3352 (26%)	Reference					
Haemoglobin (g/dL)	1003/3690 (27%)	0.90 (0.85–0.95)	< 0.001				
Gametocytaemia							
Yes	36/133 (27%)	0.86 (0.57–1.29)	0.45				
No	953/3522 (27%)	Reference					
Mixed infection							
Yes	14/25 (56%)	3.11 (1.36–7.12)	0.007	1.52 (0.52–4.46)	0.45	2.54 (1.07–5.99)	0.03
No	993/3682 (27%)	Reference		Reference		Reference	
Malaria transmission							
Low	277/726 (38%)	1.27 (0.80–2.01)	0.32				
Moderate	484/2040 (24%)	Reference					
High	246/941 (26%)	0.94 (0.60–1.47)	0.78				

Intraclass correlation, 0.06. *AAP* artesunate with atovaquone-proguanil, *AL* artemether-lumefantrine, *aOR* adjusted odds ratio, *AS* artesunate monotherapy, *ASAQ* artesunate-amodiaquine, *ASMQ* artesunate-mefloquine, *ASSP* artesunate-sulfadoxine-pyrimethamine, *BMI* body mass index, *CI* confidence interval, *DP* dihydroartemisinin-piperaquine, *EGA* estimated gestational age, *G* gravidity, *HIV* human immunodeficiency virus, *MI* multiple imputation, *OR* odds ratio, *Q* quinine monotherapy, *QC* quinine with clindamycin

### Placental malaria

Among 3033 women in four studies that assessed placental histopathology, 441 (pooled prevalence 17.6%, 95% CI 12.1 to 23.8, *I*^2^ 94%) women were categorized as no infection, 49 (1.6%, 95% CI 0.8 to 2.5, *I*^2^ 60%) acute infection, 1816 (57.5%, 95% CI 53.6 to 61.5, *I*^2^ 77%) past infection, and 727 (21.6%, 95% CI 15.9 to 27.8, *I*^2^ 93%) chronic infection. Overall pooled positivity of malaria pigment was 80.1% (2543/3033, 95% CI 73.5 to 85.9, *I*^2^ 84%). The risk factors for deposition of malaria pigment (i.e. past and chronic infection) were investigated in 2987 women.

Six antimalarial treatments were included in this analysis: AL (*n* = 893), ASAQ (*n* = 649), ASMQ (*n* = 668), DP (*n* = 658), AS (*n* = 85), and quinine (*n* = 80). Baseline characteristics (Additional Table 4) were similar to pregnant women who were included in the previous analyses.

In both the univariable and multivariable analyses, the risk of malaria pigment in the placenta did not differ between AL and the other treatments (Table 5). In the multivariable analysis, shorter interval from malaria episode to delivery (aOR 0.97 per week, 95% CI 0.95 to 0.99, *p* = 0.002), lower age (aOR 0.93 per year, 95% CI 0.91 to 0.95, *p* < 0.001), higher body temperature (aOR 1.22 per °C, 95% CI 1.00 to 1.49, *p* = 0.05), lower haemoglobin level (aOR 0.72 per g/dL, 95% CI 0.65 to 0.78, *p* < 0.001), higher baseline parasitaemia (aOR 1.67 per 10-fold increase, 95% CI 1.42 to 1.96, *p* < 0.001), and gametocytaemia before treatment (aOR 3.62, 95% CI 1.58 to 8.26, *p* = 0.002) were associated with a higher risk of the presence of malaria pigment in the placenta. When the effect of parity was analysed by different malaria transmission intensities, the adjusted risk of placental malaria in pregnant women with ≥ 2 parities was lower than nulliparous women in moderate (aOR 0.52, 95% CI 0.34 to 0.80, *p* = 0.003) and high (aOR 0.50, 95% CI 0.26 to 0.98, *p* = 0.04) transmission areas, but not in low transmission areas (aOR 1.26, 95% CI 0.62 to 2.59, *p* = 0.53) (Additional Table 5).

**Table 5:** Univariable and multivariable logistic regression on the risk of malaria pigment deposition in the placenta

Baseline characteristic	Proportion (%)	Univariable		Multivariable	
		OR (95% CI)	*p* value	aOR (95% CI)	*p* value
Treatment					
AL	733/880 (83%)	Reference		Reference	
AS	56/84 (67%)	1.63 (0.86–3.10)	0.13	1.77 (0.85–3.68)	0.13
ASAQ	544/645 (84%)	0.93 (0.67–1.29)	0.68	0.90 (0.63–1.28)	0.55
ASMQ	560/658 (85%)	0.80 (0.57–1.12)	0.20	0.78 (0.54–1.13)	0.20
DP	559/642 (87%)	0.96 (0.67–1.36)	0.81	0.91 (0.62–1.34)	0.64
Q	53/78 (68%)	1.05 (0.55–2.00)	0.89	0.80 (0.38–1.66)	0.55
Interval from malaria to delivery (week)	2505/2987 (84%)	0.98 (0.96–0.99)	0.005	0.97 (0.95–0.99)	0.002
Age (year)	2505/2987 (84%)	0.89 (0.88–0.91)	< 0.001	0.93 (0.91–0.95)	< 0.001
Parity					
0	1199/1292 (93%)	Reference		Reference	
1	498/580 (86%)	0.50 (0.36–0.69)	< 0.001	0.82 (0.58–1.17)	0.27
≥ 2	807/1112 (73%)	0.21 (0.16–0.27)	< 0.001	0.59 (0.41–0.86)	0.006
Weight (kg)	2505/2987 (84%)	0.97 (0.96–0.98)	< 0.001		
Height (cm)	2383/2806 (85%)	0.98 (0.96–1.00)	0.01		
BMI (kg/m^2^)	2383/2806 (85%)	0.95 (0.92–0.99)	0.008		
HIV infection					
Yes	18/24 (75%)	1.06 (0.40–2.82)	0.91		
No	2035/2414 (84%)	Reference			
Parasitaemia (log_10_/μL)	2505/2987 (84%)	2.04 (1.76–2.35)	< 0.001	1.67 (1.42–1.96)	< 0.001
Body temperature (°C)	2504/2986 (84%)	1.35 (1.13–1.61)	< 0.001	1.22 (1.00–1.49)	0.045
Haemoglobin (g/dL)	2500/2978 (84%)	0.65 (0.60–0.70)	< 0.001	0.72 (0.65–0.78)	< 0.001
Gametocytaemia					
Yes	84/91 (92%)	3.60 (1.62–7.97)	0.002	3.62 (1.58–8.26)	0.002
No	2417/2891 (84%)	Reference		Reference	
Mixed infection					
Yes	12/16 (75%)	1.82 (0.57–5.82)	0.31		
No	2493/2971 (84%)	Reference			
Malaria transmission					
Low	159/249 (64%)	1.16 (0.57–2.34)	0.69	0.81 (0.34–1.91)	0.63
Moderate	1588/1838 (86%)	Reference		Reference	
High	758/900 (84%)	1.14 (0.66–1.96)	0.65	1.16 (0.63–2.13)	0.64

Intraclass correlation, 0.14. *AL* artemether-lumefantrine, *aOR* adjusted odds ratio, *AS* artesunate monotherapy, *ASAQ* artesunate-amodiaquine, *ASMQ* artesunate-mefloquine, *BMI* body mass index, *CI* confidence interval, *DP* dihydroartemisinin-piperaquine, *EGA* estimated gestational age, *G* gravidity, *HIV* human immunodeficiency virus, *OR* odds ratio, *Q* quinine monotherapy

## Discussion

This IPD meta-analysis demonstrated that among the four commonly used ACTs, namely AL, ASAQ, ASMQ, and DP, the adjusted risks of stillbirth, PTB, SGA, and past or chronic placental malaria (deposition of malaria pigment in the placenta) were not different following treatment for patent microscopic falciparum malaria (uncomplicated or asymptomatic) mainly in the second or third trimester of pregnancy. The results for the other treatments (ASSP, AS, AAP, quinine, and QC) need careful interpretation because of the small number of women included in the analyses, but generally, they were associated with similar risks to AL.

The very low proportion of pregnancy loss from miscarriage observed in this pooled analysis is a consequence of the natural history of miscarriage, which decreases considerably after the end of the first trimester; as the mean gestation at enrolment was > 24.0 weeks, most participants were already beyond the high risk period for miscarriage. Therefore, the result of this analysis does not necessarily reflect the overall impact of malaria and antimalarial treatment on miscarriage, and likely underestimates it.

The low proportion of stillbirth (1.7%) in women treated with highly effective ACTs is notable compared to a previous systematic review and meta-analysis by Moore et al., reporting up to 1 in 5 stillbirths attributed to *P. falciparum* malaria infection in sub-Saharan Africa [1]. The included studies in this IPD meta-analysis screened and detected actively, and treated women for malaria (and anaemia), which is a higher level of care than what is usually provided, likely improving outcomes since early detection, effective treatment, and active follow-up for at least several weeks are beneficial. The previous report by Moore et al. [1] was derived predominantly from cross-sectional surveys, retrospective analysis, or cohort studies, before widespread uptake of early diagnosis, ACT treatment during pregnancy, and more reliable EGA measurement.

In this pooled analysis, the prevalence of SGA was higher than generalized regional estimates reported in the literature [39]. This higher prevalence of SGA in this pooled analysis of pregnant women treated for falciparum malaria was likely due to the malaria infection itself [3] despite a low proportion of febrile cases on admission. Furthermore, higher parasite density at baseline was associated with higher risk of SGA even after adjusting for gravidity/parity. This highlights the need for highly effective malaria preventive measures to lower the risk of SGA. In this study, the risk of SGA was higher with infection in the early second trimester and possibly in the late third trimester as was reported previously [3, 40, 41]. This is compatible with gestational physiology: development of the placenta is affected by malaria in early pregnancy [41, 42], and foetal weight gain increases in the final weeks before delivery [15].

The prevalence of PTB in this pooled analysis was similar to the general background population [43], suggesting that highly effective antimalarial treatment alongside improved acute care can lower the immediate risk of PTB. The rate of febrile symptomatic infections in this pooled analysis was low which maybe another reason for relatively low prevalence of PTB. In contrast to SGA, in the second and third trimester, the risk of PTB from malaria infection increases towards term as was shown in an observational cohort study comparing women with or without malaria infection [3]. Although there were only a few cases of first trimester malaria infection included in this analysis, the current study suggests that treated malaria infections in the first trimester are associated with an increased risk of PTB, possibly mediated by impaired placental development [44].

The prevalence of pigmentation in the placenta in this pooled analysis was higher than the prevalence reported in the general population in malaria-endemic areas [45], which is explained by the fact that all included women had peripheral malaria detected and treated during pregnancy. The pooled proportion of pigment deposition was 80.1%, a reminder that placental malaria is a proxy for malaria in pregnancy. Placental malaria is reported to be associated with higher risks of LBW and PTB [16, 18–20, 46]. PTB is more likely to be caused by an active infection at delivery (i.e. acute and chronic infection of the placenta), whereas previous infection (i.e. past and chronic infection) is associated with a higher risk of LBW or IUGR [16, 19, 20]. The risk of placental malaria is, in turn, affected by gravidity, age, number of malaria infections, and parasite density [16, 47], which was confirmed in this IPD meta-analysis (with the exception of frequency of parasitaemia, which could not be assessed). Additionally, a higher risk of placental malaria was observed in women with higher body temperature before treatment, which can be a surrogate for the severity of the infection; inflammatory cytokines causing fever (e.g. TNF-α) are reported to be associated with a higher parasite burden in the placenta [16, 48]. The presence of gametocytes at baseline and lower haemoglobin can reflect a more extended period of infection before treatment [49, 50], although haemoglobin in pregnancy can be affected by gestational age or provision of supplements at antenatal care. A longer interval between the treated malaria episode and delivery was associated with a lower risk of placental malaria, which has also been shown in previous studies [46, 51–53]. These results may support an earlier finding from a low transmission area that malaria pigment in the placenta can be cleared over time after prompt and effective treatment or that early detection and treatment prevents pigment depositing in the placenta [53–56].

There are several strengths of this meta-analysis. Primarily, use of IPD, collected in prospective trials in which gestational age was predominantly assessed by ultrasound, allowed analysis of the largest number of pregnant women ever to be assembled to assess the impact of different treatments by the gestation age of falciparum malaria infection in pregnancy. With no registered trials currently recruiting pregnant women for assessment of efficacy of antimalarial treatment in pregnancy [23], it is unlikely that a larger dataset will be available in the foreseeable future. Gestational age was strictly defined and assessed with ultrasound for > 90% of women included. Individual studies used different cut-offs or definitions [11]; thus, aggregated data meta-analysis was difficult. With IPD, standardized definitions for both exposures and outcomes were applied. SGA was assessed using the same standard growth chart, and the use of SGA, rather than LBW or birthweight, is preferable as it is a more reliable indicator of foetal growth.

Some limitations of this study should be considered. Though confounders for each study were assessed (e.g. age, height, BMI, parity or gravidity, HIV status) and adjusted in our IPD meta-analyses, it is possible that some potential confounders remained unadjusted. For example, the number of malaria episodes during pregnancy was reported to be cumulatively associated with an increased risk of SGA [3, 5] or placental malaria [46]. The use of insecticide-treated bed nets and the history of malaria and antimalarial use including IPTp before and after the study period were not always available (or not systematically collected). As IPTp is associated with a lower risk of LBW [57], it is possible that we have underestimated the impact of malaria on the pregnancy outcomes by missing some unreported IPTp intervention [11]. However, these two preventive measures can be regarded as an unmeasured study-level information and was statistically taken into account by mixed-effects model. Most of the women included in this pooled analysis were not febrile and had relatively low parasitaemia, and this might be used to explain the lack of a difference between highly efficacious antimalarial treatments. This characteristic is, however, more likely to underestimate the adverse impact by malaria. The high risk of SGA after treated malaria in this pooled analysis is profound and supports a policy of prevention but also promotes the use of early detection by screening and treatment of parasitaemia regardless of symptoms for pregnancy. Previous systematic reviews concluded that malaria in pregnancy is associated with increased risk of stillbirth, LBW, and PTB compared with those without malaria [1, 4]. The current study, including only malaria treatment episodes, compared the impact of different treatments, and we have additionally compared them with the most recent regional estimates. Although it was not possible to assess the risks of outcomes without treatment, with what we now know about the harmful effects of malaria, such a study would clearly be unethical. Similarly, the first documented diagnosis of malaria does not exclude previous malaria episodes: included women may have had malaria before study enrolment, and late study enrolment might mean antenatal care attendance started in later gestation.

Multiple factors, including obstetric history, morbidity such as hypertensive disorders of pregnancy, gestational diabetes, sub-optimal gestational weight gain, and perinatal depression, and social factors, including employment, smoking, marital status, and literacy, can all be associated with pregnancy outcomes [43, 58–62], but this information was not available in most studies [11]. The previous history of pregnancy loss (stillbirth and miscarriage) was only roughly estimated by parity and gravidity in this meta-analysis. BMI was assessed using body weight at enrolment rather than pre-pregnancy weight. For comparability of outcomes to previous studies, infants with congenital abnormality and twins were excluded from our analyses. This exclusion would underestimate the true burden of stillbirth, PTB, and SGA and may reduce the effect size of comparisons but not the direction of associations. The number of twins and congenital abnormalities was small and did not differ by antimalarial treatment. As less than 1% of the pregnant women included in this analysis were enrolled and treated in the first trimester, the risk of congenital abnormality due to antimalarial drugs is expected to be very low in the current study. Congenital abnormalities in stillborns were presumably underreported, but autopsy or chromosomal investigation was not mentioned in any of the study sites [63, 64]. Due to the availability of information, placental pigmentation was analysed only qualitatively, while a previous study revealed AL reduced the level of pigmentation more than quinine [53]. Quantitative analysis can be a better way to assess the impact of different treatments, particularly regarding the clearance (rather than prevalence) of placental malaria. Antimalarial resistance can affect the response to treatment in terms of efficacy which impacts pregnancy outcomes, and needs to be addressed in the future, considering in particular the expansion of artemisinin resistance.

Although the use of artemisinin derivatives in the first trimester seems to be safe [2, 65], the small number of pregnant women available in this analysis is not sufficient to compare the safety profile of the different ACTs in the first trimester. The risk of PTB following malaria infection in the first trimester can be higher than infection in later gestational period, indicating that prevention measures should start early in pregnancy. Evidence on the efficacy and safety of ACT treatment in the first trimester, either as treatment or prevention, is thus needed.

## Conclusions

This IPD meta-analysis of over 4500 women infected with falciparum malaria from both Asia and sub-Saharan Africa demonstrated that the risks of late miscarriage and stillbirth were low, and late miscarriage, stillbirth, PTB, SGA, and placental malaria were not different between the most commonly used ACTs (i.e. AL, ASAQ, ASMQ, and DP) in the second and third trimesters. Accurate assessment of EGA, malaria episodes beyond the current study framework (28–63 days), and thorough reporting of congenital abnormality, including abnormality in stillbirth, should be considered in future studies. Early diagnosis of parasitaemia, regardless of symptoms, and treatment with highly effective ACTs may result in improved pregnancy outcomes, but highly effective preventive measures are required to reduce the high rate of SGA.

## Supplementary information

**Additional file 1.** Methods of multiple imputation.

**Additional file 2.** Risk of bias assessment.

**Additional file 3.** Sensitivity analysis.

**Additional file 4: Additional Figure 1.** Forest plot of the proportion of miscarriage for each study site.

**Additional file 5: Additional Figure 2.** Forest plot of the proportion of stillbirth for each study site.

**Additional file 6: Additional Figure 3.** Forest plot of the proportion of moderate-to-late preterm birth for each study site.

**Additional file 7: Additional Figure 4.** Forest plot of the proportion of small-for-gestational-age for each study site.

**Additional file 8: Additional Table 1.** Summary of the studies included in the pooled analyses.

**Additional file 9: Additional Table 2.** Baseline characteristics of pregnant women assessed for moderate-to-late preterm birth.

**Additional file 10: Additional Table 3.** Baseline characteristics of pregnant women assessed for small-for-gestational-age.

**Additional file 11: Additional Table 4.** Baseline characteristics of pregnant women assessed for deposition of malaria pigment in the placenta.

**Additional file 12: Additional Table 5.** Multivariable logistic regression on the risk of deposition of malaria pigment in the placenta by parity in different malaria transmission area.

## Data Availability

The datasets supporting the conclusions of this article are available from the WWARN data repository (http://www.wwarn.org/working-together/sharing-data/accessing-data) for researchers who meet the criteria for access to confidential data.

## Abbreviations

AAP: Artesunate with atovaquone-proguanil
ACTs: Artemisinin-based combination therapies
AL: Artemether-lumefantrine
aOR: Adjusted odds ratio
AS: Artesunate monotherapy
ASAQ: Artesunate-amodiaquine
ASMQ: Artesunate-mefloquine
ASSP: Artesunate-sulfadoxine-pyrimethamine
BMI: Body mass index
DP: Dihydroartemisinin-piperaquine
EGA: Estimated gestational age
G: Gravidity
HIV: Human immunodeficiency virus
IPD: Individual patient data
IPTp: Intermittent preventive treatment in pregnancy
IUGR: Intrauterine growth restriction
LBW: Low birthweight
MI: Multiple imputation
PTB: Preterm birth
OR: Odds ratio
Q: Quinine monotherapy
QC: Quinine with clindamycin
SD: Standard deviation
SGA: Small for gestational age
SP: Sulfadoxine-pyrimethamine
WWARN: WorldWide Antimalarial Resistance Network
