# Supplementary material for: Pregnancy outcomes and risk of placental malaria after artemisinin-based and quinine-based treatment for uncomplicated falciparum malaria in pregnancy: a WorldWide Antimalarial Resistance Network systematic review and individual patient data meta-analysis
Source: BMC Med. 2020 Jun 2;18:138. doi: 10.1186/s12916-020-01592-z (PMC7263905; doi:10.1186/s12916-020-01592-z)
Supplement: Supplementary file 1 — Additional file 1. Methods of multiple imputation. [file 12916_2020_1592_MOESM1_ESM.pdf]

Additional File 1. Methods of multiple imputation

Multiple imputation was conducted because height (and BMI), which are known factors associated with pregnancy outcomes, were missing up to 15%. HIV status was also missing in up to 18%, even when the pregnant women on the Thailand-Myanmar border (where seroprevalence of HIV was <0·5%) were regarded as negative.

All variables assessed in the univariable model and a variable for study site (as a fixed variable) were included in the imputation model under fully conditional specification with data augmentation for perfect prediction. BMI was passively calculated based on the imputed height and weight. Imputation was conducted for each outcome. The variable for outcome were also included in each imputation model.

Because missingness of height was more frequent in quinine and quinine with clindamycin, complete case analyses were biased when adjusted for height. The multivariable results by multiple imputation method were approximately similar to univariable results by complete case analyses method.
