# Supplementary material for: Pregnancy outcomes and risk of placental malaria after artemisinin-based and quinine-based treatment for uncomplicated falciparum malaria in pregnancy: a WorldWide Antimalarial Resistance Network systematic review and individual patient data meta-analysis
Source: BMC Med. 2020 Jun 2;18:138. doi: 10.1186/s12916-020-01592-z (PMC7263905; doi:10.1186/s12916-020-01592-z)
Supplement: Supplementary file 3 — Additional file 3. Sensitivity analysis. [file 12916_2020_1592_MOESM3_ESM.pdf]

Additional File 3. Sensitivity analysis

In some studies, estimated gestational age (EGA) in weeks (without days) was available at malaria episodes but not at delivery. When the EGA at delivery was calculated from the EGA at malaria episode, the difference of a maximum of seven days (i.e. day 0 or day 6 of the same week) can affect the binary category of PTB and SGA. For those studies, results assuming day 0 of the week were presented as the primary analyses, and those assuming day 6 of the week were presented as sensitivity analyses.

For PTB, the overall pooled proportion of extremely PTB (<28.0 weeks), very PTB (≥28 to <32.0 weeks) and moderate to late PTB (≥32.0 to <37.0 weeks) was 0·0% (5/2610, 95% CI 0·0 to 0·0, I^2 0%), 0·1% (34/3279, 95% CI 0·0 to 0·4, I^2 0%) and 7·8% (438/4147, 95% CI 5·6 to 10·3, I^2 81%), respectively. The results of the univariable and multivariable analyses were similar to the primary analysis results (Additional Table 3-1), except that malaria transmission intensity was not associated with the risk of PTB.

For SGA, the overall pooled proportion of SGA was 38·1% (1310/3674, 95% CI 32·7 to 43·6, I^2 88%). The results of the univariable and multivariable analyses were similar to the primary analysis results (Additional Table 3-2).

Additional Table 3-1. Univariable and multivariable logistic regression on the risk of moderate-to-late preterm birth assuming day 6 on the week

Baseline characteristic		Proportion (%)	Univariable		Multivariable (Complete case)		Multivariable (MI)	
			OR (95% CI)	p-value	aOR (95% CI)	p-value	aOR (95% CI)	p-value
Treatment								
AL		114/1043 (11%)	Reference		Reference		Reference	
AAP		4/76 (5%)	0.62 (0.19–2.01)	0.42	1.77 (0.33–9.45)	0.51	0.55 (0.16–1.89)	0.34
AS		15/174 (9%)	1.57 (0.72–3.40)	0.26	1.58 (0.61–4.12)	0.35	1.27 (0.55–2.91)	0.57
ASAQ		82/750 (11%)	0.95 (0.69–1.31)	0.77	1.03 (0.71–1.49)	0.87	0.97 (0.70–1.34)	0.84
ASMQ		81/927 (9%)	0.95 (0.69–1.32)	0.77	0.78 (0.52–1.16)	0.21	0.91 (0.65–1.28)	0.59
ASSP		21/148 (14%)	1.81 (0.88–3.71)	0.11	0.89 (0.36–2.20)	0.80	1.67 (0.78–3.58)	0.19
DP		102/807 (13%)	1.19 (0.88–1.61)	0.26	1.20 (0.87–1.67)	0.27	1.14 (0.83–1.56)	0.42
Q		15/171 (9%)	1.28 (0.63–2.59)	0.50	2.40 (0.59–9.71)	0.22	1.53 (0.72–3.23)	0.27
QC		4/51 (8%)	1.18 (0.32–4.44)	0.80	No data		1.18 (0.29–4.81)	0.82
EGA at malaria episode								
4.0–13.9		5/38 (13%)	4.34 (1.56–12.06)	0.005	2.71 (0.74–9.91)	0.13	4.02 (1.41–11.43)	0.009
14.0–19.9		53/866 (6%)	Reference		Reference		Reference	
20.0–23.9		101/1029 (10%)	1.86 (1.31–2.64)	0.001	1.78 (1.20–2.65)	0.004	1.85 (1.30–2.65)	<0.001
24.0–27.9		90/921 (10%)	1.98 (1.38–2.84)	<0.001	2.31 (1.54–3.46)	<0.001	2.17 (1.50–3.14)	<0.001
28.0–36.9 (weeks)		189/1293 (15%)	3.79 (2.71–5.31)	<0.001	4.80 (3.26–7.06)	<0.001	4.51 (3.19–6.38)	<0.001
Age Group								
<20		210/1334 (16%)	Reference		Reference		Reference	
20–25		106/1333 (8%)	0.50 (0.39–0.65)	<0.001	0.61 (0.43–0.86)	0.005	0.60 (0.44–0.82)	0.001
25–30		68/804 (8%)	0.54 (0.40–0.73)	<0.001	0.91 (0.56–1.48)	0.70	0.73 (0.48–1.13)	0.16
30–35		28/401 (7%)	0.44 (0.29–0.67)	<0.001	0.52 (0.27–0.98)	0.04	0.56 (0.33–0.97)	0.04
≥35 (years)		26/275 (9%)	0.71 (0.46–1.11)	0.14	0.77 (0.37–1.58)	0.48	0.92 (0.52–1.63)	0.78
Pregnancy history								
G 1		210/1449 (14%)	Reference		Reference		Reference	
G 2 with no loss		58/706 (8%)	0.55 (0.41–0.75)	<0.001	0.72 (0.48–1.07)	0.10	0.68 (0.48–0.98)	0.04
G≥3 with no loss		91/1129 (8%)	0.54 (0.41–0.70)	<0.001	0.60 (0.37–0.96)	0.03	0.66 (0.44–1.00)	0.048
G 2 with 1 loss		27/252 (11%)	0.81 (0.52–1.26)	0.35	1.12 (0.70–1.80)	0.64	0.92 (0.58–1.45)	0.72
G≥3 with 1 loss		34/436 (8%)	0.56 (0.38–0.82)	0.003	0.64 (0.38–1.10)	0.11	0.66 (0.41–1.07)	0.09
G≥3 with ≥2 losses		15/138 (11%)	1.01 (0.57–1.79)	0.97	1.38 (0.67–2.84)	0.38	1.16 (0.61–2.21)	0.64
Weight (kg)		437/4146 (11%)	0.98 (0.97–1.00)	0.009				
Height (cm)		399/3670 (11%)	0.98 (0.97–1.00)	0.05	0.98 (0.96–1.00)	0.08	0.98 (0.96–1.00)	0.05
BMI (kg/m^2)		399/3670 (11%)	0.96 (0.92–1.00)	0.047	0.95 (0.91–1.00)	0.04	0.94 (0.9–0.99)	0.009
HIV infection	Yes	4/31 (13%)	1.97 (0.65–5.98)	0.23	2.95 (0.54–16.03)	0.21	1.43 (0.43–4.8)	0.55
	No	376/3476 (11%)	Reference		Reference		Reference	
Parasitaemia (log_{10}/μL)		438/4147 (11%)	1.08 (0.95–1.22)	0.23				
Fever >37.5°C	Yes	35/375 (9%)	1.08 (0.74–1.57)	0.70				
	No	393/3734 (11%)	Reference					
Haemoglobin (g/dL)		435/4123 (11%)	0.93 (0.86–1.00)	0.05				
Gametocytaemia	Yes	21/154 (14%)	1.68 (1.03–2.72)	0.04				
	No	407/3936 (10%)	Reference					
Mixed Infection	Yes	2/29 (7%)	1.09 (0.25–4.71)	0.91				
	No	436/4118 (11%)	Reference					
Malaria transmission								
Low		72/929 (8%)	0.81 (0.47–1.41)	0.46				
Moderate		265/2247 (12%)	Reference					
High		101/971 (10%)	0.92 (0.49–1.71)	0.79				

Intraclass correlation: 0.09. AAP: artesunate with atovaquone-proguanil, AL: artemether-lumefantrine, aOR: adjusted odds ratio, AS: artesunate monotherapy, ASAQ: artesunate-amodiaquine, ASMQ: artesunate-mefloquine, ASSP: artesunate-sulfadoxine-pyrimethamine, BMI: body mass index, CI: confidence interval, DP: dihydroartemisinin-piperaquine, EGA: estimated gestational age, G: gravidity, HIV: human immunodeficiency virus, OR: odds ratio, Q: quinine monotherapy, QC: quinine with clindamycin.

Additional Table 3-2. Univariable and multivariable logistic regression on the risk of small-for-gestational-age assuming day 6 on the week

Baseline characteristic		Proportion (%)	Univariable		Multivariable (Complete case)		Multivariable (MI)	
			OR (95% CI)	p-value	aOR (95% CI)	p-value	aOR (95% CI)	p-value
Treatment								
AL		307/969 (32%)	Reference		Reference		Reference	
AAP		25/55 (45%)	1.61 (0.78–3.29)	0.20	0.52 (0.14–1.92)	0.33	1.43 (0.60–3.39)	0.42
AS		56/154 (36%)	1.22 (0.75–1.98)	0.42	1.20 (0.68–2.14)	0.53	1.16 (0.68–1.98)	0.58
ASAQ		253/695 (36%)	1.24 (0.99–1.56)	0.06	1.13 (0.86–1.49)	0.39	1.28 (1.01–1.62)	0.045
ASMQ		337/810 (42%)	1.11 (0.89–1.39)	0.35	1.09 (0.84–1.42)	0.51	1.10 (0.87–1.38)	0.44
ASSP		67/128 (52%)	1.51 (0.92–2.49)	0.10	1.77 (0.98–3.22)	0.06	1.39 (0.81–2.39)	0.23
DP		230/703 (33%)	0.98 (0.77–1.23)	0.84	0.98 (0.76–1.27)	0.87	0.97 (0.76–1.24)	0.83
Q		20/119 (17%)	0.50 (0.27–0.89)	0.02	0.16 (0.04–0.65)	0.01	0.56 (0.28–1.12)	0.10
QC		15/41 (37%)	1.09 (0.46–2.58)	0.84	No data		0.89 (0.33–2.46)	0.83
EGA at malaria episode								
4.0–13.9		10/31 (32%)	0.66 (0.29–1.49)	0.32	0.65 (0.26–1.60)	0.35	0.59 (0.25–1.36)	0.21
14.0–19.9		288/758 (38%)	Reference		Reference		Reference	
20.0–23.9		334/911 (37%)	0.84 (0.68–1.03)	0.10	0.84 (0.66–1.07)	0.16	0.84 (0.68–1.04)	0.11
24.0–27.9		295/816 (36%)	0.75 (0.61–0.94)	0.01	0.80 (0.62–1.04)	0.09	0.81 (0.65–1.02)	0.07
28.0–36.9		367/1119 (33%)	0.58 (0.47–0.72)	<0.001	0.64 (0.50–0.82)	<0.001	0.67 (0.54–0.83)	<0.001
≥37.0 (weeks)		16/39 (41%)	1.03 (0.51–2.05)	0.94	0.47 (0.12–1.81)	0.27	1.28 (0.62–2.64)	0.51
Age Group								
<20		494/1199 (41%)	Reference					
20–25		431/1175 (37%)	0.69 (0.58–0.83)	<0.001				
25–30		210/710 (30%)	0.50 (0.40–0.61)	<0.001				
30–35		96/348 (28%)	0.45 (0.34–0.59)	<0.001				
≥35 (years)		79/242 (33%)	0.56 (0.41–0.76)	<0.001				
Pregnancy history								
G 1		577/1312 (44%)	Reference		Reference		Reference	
G 2 with no loss		192/606 (32%)	0.54 (0.44–0.66)	<0.001	0.59 (0.46–0.75)	<0.001	0.57 (0.46–0.70)	<0.001
G≥3 with no loss		257/998 (26%)	0.40 (0.33–0.48)	<0.001	0.47 (0.37–0.59)	<0.001	0.47 (0.38–0.57)	<0.001
G 2 with 1 loss		99/225 (44%)	0.80 (0.59–1.07)	0.13	0.72 (0.51–1.01)	0.06	0.79 (0.59–1.08)	0.14
G≥3 with 1 loss		116/379 (31%)	0.46 (0.36–0.59)	<0.001	0.52 (0.39–0.71)	<0.001	0.54 (0.41–0.70)	<0.001
G≥3 with ≥2 losses		50/117 (43%)	0.77 (0.52–1.14)	0.19	0.77 (0.47–1.25)	0.29	0.81 (0.54–1.22)	0.31
Weight (kg)		1310/3674 (36%)	0.95 (0.94–0.96)	<0.001				
Height (cm)		1193/3315 (36%)	0.96 (0.94–0.97)	<0.001	0.95 (0.94–0.97)	<0.001	0.95 (0.94–0.96)	<0.001
BMI (kg/m^2)		1193/3315 (36%)	0.92 (0.90–0.95)	<0.001	0.92 (0.89–0.95)	<0.001	0.92 (0.90–0.95)	<0.001
HIV infection	Yes	7/24 (29%)	0.82 (0.32–2.08)	0.68	2.26 (0.55–9.23)	0.26	1.11 (0.47–2.62)	0.82
	No	225/568 (40%)	Reference		Reference		Reference	
Parasitaemia (log_{10}/µL)		1310/3674 (36%)	1.24 (1.13–1.35)	<0.001	1.16 (1.04–1.30)	0.007	1.13 (1.03–1.24)	0.01
Fever >37.5°C	Yes	124/324 (38%)	1.03 (0.81–1.33)	0.80				
	No	1178/3322 (35%)	Reference					
Haemoglobin (g/dL)		1306/3657 (36%)	0.88 (0.83–0.92)	<0.001				
Gametocytaemia	Yes	45/133 (34%)	0.91 (0.62–1.32)	0.61				
	No	1246/3490 (36%)	Reference					
Mixed Infection	Yes	14/25 (56%)	2.98 (1.30–6.80)	0.01	1.43 (0.49–4.20)	0.51	2.47 (1.05–5.82)	0.04
	No	1296/3649 (36%)	Reference		Reference		Reference	
Malaria transmission								
Low		277/726 (38%)	0.90 (0.59–1.37)	0.61				
Moderate		680/2018 (34%)	Reference					
High		353/930 (38%)	0.82 (0.54–1.25)	0.36				

Intraclass correlation: 0.06. AAP: artesunate with atovaquone-proguanil, AL: artemether-lumefantrine, aOR: adjusted odds ratio, AS: artesunate monotherapy, ASAQ: artesunate-amodiaquine, ASMQ: artesunate-mefloquine, ASSP: artesunate-sulfadoxine-pyrimethamine, BMI: body mass index, CI: confidence interval, DP: dihydroartemisinin-piperaquine, EGA: estimated gestational age, G: gravidity, HIV: human immunodeficiency virus, OR: odds ratio, Q: quinine monotherapy, QC: quinine with clindamycin.

One reviewer requested to run anlyses stratified by geographical region. We have conducted requested analyses for moderate-to-late PTB, SGA and placental malaria, which have enough number of events. In Asia, there are only one or two study sites included, so interpretation needs caution. Same imputed dataset was used for each outcome (except placental malaria which multiple imputation was not needed).

Additional Table 3-3. Multivariable logistic regression on the risk of moderate-to-late preterm birth stratified by geographical region using multiple imputation

Baseline characteristic		Proportion (%)	Multivariable (All)		Multivariable (Africa)		Multivariable (Asia)	
			aOR (95% CI)	p-value	aOR (95% CI)	p-value	aOR (95% CI)	p-value
Treatment								
AL		156/1035 (15%)	Reference		Reference		Reference	
AAP		4/76 (5%)	0.69 (0.19–2.44)	0.56	No data		1.88 (0.40-8.82)	0.43
AS		15/174 (9%)	1.49 (0.64–3.50)	0.36	No data		3.00 (0.83-10.82)	0.09
ASAQ		124/747 (17%)	1.05 (0.79–1.39)	0.75	1.03 (0.78-1.38)	0.82	No data	
ASMQ		128/926 (14%)	1.07 (0.80–1.41)	0.65	1.01 (0.75-1.35)	0.97	4.60 (1.26-16.77)	0.02
ASSP		22/147 (15%)	1.83 (0.88–3.81)	0.10	4.90 (1.23-19.48)	0.02	4.02 (1.05-15.38)	0.04
DP		151/804 (19%)	1.22 (0.93–1.60)	0.15	1.23 (0.93-1.62)	0.15	1.23 (0.19-7.88)	0.83
Q		15/171 (9%)	1.67 (0.79–3.54)	0.18	1.74 (0.56-5.44)	0.34	5.85 (1.46-23.53)	0.01
QC		4/51 (8%)	1.39 (0.34–5.67)	0.65	No data		2.97 (0.62-14.21)	0.17
EGA at malaria episode								
4.0–13.9		5/38 (13%)	3.92 (1.38–11.13)	0.01	3.75 (0.34-40.99)	0.28	2.89 (0.81-10.30)	0.10
14.0–19.9		78/863 (9%)	Reference		Reference		Reference	
20.0–23.9		136/1023 (13%)	1.71 (1.25–2.32)	<0.001	1.84 (1.33-2.56)	<0.001	1.04 (0.42-2.57)	0.94
24.0–27.9		142/917 (15%)	2.37 (1.74–3.23)	<0.001	2.53 (1.81-3.53)	<0.001	1.78 (0.75-4.24)	0.19
28.0–36.9 (weeks)		258/1290 (20%)	4.33 (3.21–5.84)	<0.001	5.00 (3.61-6.92)	<0.001	1.86 (0.86-4.04)	0.11
Age Group								
<20		284/1323 (21%)	Reference		Reference		Reference	
20–25		154/1330 (12%)	0.67 (0.51–0.87)	0.003	0.68 (0.51-0.91)	0.01	0.57 (0.26-1.23)	0.15
25–30		100/803 (12%)	0.82 (0.56–1.18)	0.29	0.93 (0.62-1.40)	0.73	0.50 (0.19-1.33)	0.16
30–35		46/401 (11%)	0.72 (0.45–1.12)	0.15	0.79 (0.48-1.31)	0.37	0.45 (0.15-1.38)	0.16
≥35 (years)		35/274 (13%)	0.99 (0.60–1.64)	0.98	1.28 (0.74-2.24)	0.38	0.40 (0.12-1.37)	0.14
Pregnancy history								
G 1		284/1439 (20%)	Reference		Reference		Reference	
G 2 with no loss		81/704 (12%)	0.65 (0.48–0.89)	0.007	0.64 (0.46-0.90)	0.01	0.63 (0.26-1.51)	0.30
G≥3 with no loss		141/1128 (12%)	0.67 (0.47–0.96)	0.03	0.57 (0.39-0.85)	0.01	1.48 (0.63-3.50)	0.37
G 2 with 1 loss		41/251 (16%)	0.95 (0.65–1.40)	0.81	0.96 (0.64-1.45)	0.86	0.90 (0.25-3.27)	0.87
G≥3 with 1 loss		51/435 (12%)	0.66 (0.44–0.99)	0.046	0.62 (0.40-0.97)	0.03	0.74 (0.24-2.25)	0.59
G≥3 with ≥2 losses		18/137 (13%)	1.00 (0.56–1.79)	1.00	0.62 (0.29-1.36)	0.24	2.79 (1.04-7.47)	0.04
Weight (kg)		618/4130 (15%)						
Height (cm)		579/3655 (16%)	0.98 (0.96–0.99)	0.01	0.98 (0.96-1.00)	0.02	0.98 (0.93-1.04)	0.52
BMI (kg/m^2)		579/3655 (16%)	0.94 (0.90–0.97)	<0.001	0.94 (0.90-0.98)	0.002	0.94 (0.84-1.06)	0.32
HIV infection	Yes	4/31 (13%)	1.22 (0.36–4.09)	0.75	1.10 (0.37-3.24)	0.87	No data	
	No	527/3462 (15%)	Reference		Reference			
Parasitaemia (log_{10}/µL)		619/4131 (15%)						
Fever >37.5°C	Yes	45/375 (12%)						
	No	563/3719 (15%)						
Haemoglobin (g/dL)		616/4107 (15%)						
Gametocytaemia	Yes	24/153 (16%)						
	No	584/3922 (15%)						
Mixed Infection	Yes	2/29 (7%)						
	No	617/4102 (15%)						
Malaria transmission								
Low		72/929 (8%)	0.42 (0.22–0.81)	0.009	0.84 (0.26-2.69)	0.77		
Moderate		397/2236 (18%)	Reference		Reference		No data	
High		150/966 (16%)	0.86 (0.47–1.58)	0.63	0.69 (0.34-1.42)	0.31	No data	

AAP: artesunate with atovaquone-proguanil, AL: artemether-lumefantrine, aOR: adjusted odds ratio, AS: artesunate monotherapy, ASAQ: artesunate-amodiaquine, ASMQ: artesunate-mefloquine, ASSP: artesunate-sulfadoxine-pyrimethamine, BMI: body mass index, CI: confidence interval, DP: dihydroartemisinin-piperaquine, EGA: estimated gestational age, G: gravidity, HIV: human immunodeficiency virus, MI: multiple imputation, OR: odds ratio, Q: quinine monotherapy, QC: quinine with clindamycin.

Additional Table 3-4. Multivariable logistic regression on the risk of small-for-gestational-age stratified by geographical region using multiple imputation

Baseline characteristic	Proportion (%)	Multivariable (All)		Multivariable (Africa)		Multivariable (Asia)	
		aOR (95% CI)	p-value	aOR (95% CI)	p-value	aOR (95% CI)	p-value
Treatment							
AL	241/973 (25%)	Reference		Reference		Reference	
AAP	25/55 (45%)	1.85 (0.72–4.75)	0.20	No data		2.19 (1.05-4.53)	0.04
AS	56/154 (36%)	1.26 (0.74–2.16)	0.40	No data		1.41 (0.80-2.50)	0.24
ASAQ	164/700 (23%)	1.05 (0.80–1.37)	0.73	1.04 (0.79-1.36)	0.79	No data	
ASMQ	257/820 (31%)	1.00 (0.78–1.28)	0.99	1.01 (0.78-1.31)	0.92	2.00 (1.10-3.67)	0.02
ASSP	66/129 (51%)	1.37 (0.79–2.36)	0.27	0.29 (0.07-1.19)	0.09	3.21 (1.71-6.02)	<0.001
DP	163/716 (23%)	0.87 (0.67–1.14)	0.31	0.86 (0.65-1.13)	0.27	1.99 (0.84-4.70)	0.12
Q	20/119 (17%)	0.69 (0.32–1.52)	0.36	0.67 (0.27-1.68)	0.39	0.85 (0.35-2.07)	0.72
QC	15/41 (37%)	1.11 (0.37- 3.35)	0.86	No data		1.37 (0.61-3.08)	0.44
EGA at malaria episode							
4.0–13.9	10/31 (32%)	0.62 (0.27–1.45)	0.27	0.14 (0.01-2.70)	0.19	0.82 (0.32-2.09)	0.68
14.0–19.9	227/765 (30%)	Reference		Reference		Reference	
20.0–23.9	246/914 (27%)	0.80 (0.63–1.00)	0.05	0.77 (0.60-1.00)	0.05	0.85 (0.50-1.43)	0.54
24.0–27.9	219/820 (27%)	0.80 (0.63–1.01)	0.07	0.77 (0.59-1.00)	0.05	0.88 (0.51-1.53)	0.65
28.0–36.9	289/1138 (25%)	0.70 (0.55–0.87)	0.002	0.58 (0.45-0.76)	<0.001	1.21 (0.76-1.95)	0.42
≥37.0 (weeks)	16/39 (41%)	1.36 (0.66–2.84)	0.41	0.29 (0.05-1.83)	0.19	2.81 (1.13-6.99)	0.03
Age Group							
<20	379/1202 (32%)						
20–25	332/1185 (28%)						
25–30	170/717 (24%)						
30–35	63/357 (18%)						
≥35 (years)	63/246 (26%)						
Pregnancy history							
G 1	444/1316 (34%)	Reference		Reference		Reference	
G 2 with no loss	154/614 (25%)	0.62 (0.49–0.78)	<0.001	0.55 (0.42-0.72)	<0.001	0.87 (0.54-1.39)	0.55
G≥3 with no loss	186/1011 (18%)	0.48 (0.39–0.60)	<0.001	0.46 (0.36-0.58)	<0.001	0.57 (0.36-0.90)	0.02
G 2 with 1 loss	76/225 (34%)	0.82 (0.59–1.12)	0.21	0.80 (0.56-1.13)	0.20	0.94 (0.40-2.21)	0.89
G≥3 with 1 loss	88/387 (23%)	0.54 (0.41–0.72)	<0.001	0.52 (0.37-0.72)	<0.001	0.67 (0.38-1.18)	0.16
G≥3 with ≥2 losses	40/117 (34%)	0.79 (0.51–1.21)	0.27	0.73 (0.42-1.29)	0.28	0.83 (0.41-1.67)	0.61
Weight (kg)	1007/3707 (27%)						
Height (cm)	891/3347 (27%)	0.95 (0.93–0.96)	<0.001	0.94 (0.93-0.96)	<0.001	0.96 (0.93-0.99)	0.01
BMI (kg/m^2)	891/3347 (27%)	0.92 (0.89–0.95)	<0.001	0.92 (0.89-0.96)	<0.001	0.92 (0.86-0.99)	0.03
HIV infection Yes	6/24 (25%)	1.04 (0.43–2.55)	0.92	1.22 (0.45-3.26)	0.69	No data	
No	849/3105 (27%)	Reference		Reference			
Parasitaemia (log_{10}/µL)	1007/3707 (27%)	1.14 (1.03–1.26)	0.009	1.14 (1.01-1.29)	0.03	1.10 (0.93-1.31)	0.27
Fever >37.5°C Yes	112/325 (34%)						
No	888/3352 (26%)						
Haemoglobin (g/dL)	1003/3690 (27%)						
Gametocytaemia Yes	36/133 (27%)						
No	953/3522 (27%)						
Mixed Infection Yes	14/25 (56%)	2.54 (1.07–5.99)	0.03	19.79 (1.55-252.07)	0.02	1.88 (0.73-4.86)	0.19
No	993/3682 (27%)	Reference					
Malaria transmission							
Low	277/726 (38%)						
Moderate	484/2040 (24%)						
High	246/941 (26%)						

AAP: artesunate with atovaquone-proguanil, AL: artemether-lumefantrine, aOR: adjusted odds ratio, AS: artesunate monotherapy, ASAQ: artesunate-amodiaquine, ASMQ: artesunate-mefloquine, ASSP: artesunate-sulfadoxine-pyrimethamine, BMI: body mass index, CI: confidence interval, DP: dihydroartemisinin-piperaquine, EGA: estimated gestational age, G: gravidity, HIV: human immunodeficiency virus, MI: multiple imputation, OR: odds ratio, Q: quinine monotherapy, QC: quinine with clindamycin.

Additional Table 3-5. Multivariable logistic regression on the risk of malaria pigment deposition stratified by geographical region

Baseline characteristic		Proportion (%)	Multivariable (All)		Multivariable (Africa)		Multivariable (Asia)	
			aOR (95% CI)	p-value	aOR (95% CI)	p-value	aOR (95% CI)	p-value
Treatment								
AL		733/880 (83%)	Reference		Reference		Reference	
AS		56/84 (67%)	1.77 (0.85–3.68)	0.13	No data		1.91 (0.91-4.00)	0.09
ASAQ		544/645 (84%)	0.90 (0.63–1.28)	0.55	0.90 (0.63-1.28)	0.57	No data	
ASMQ		560/658 (85%)	0.78 (0.54–1.13)	0.20	0.80 (0.55-1.15)	0.23	No data	
DP		559/642 (87%)	0.91 (0.62–1.34)	0.64	0.91 (0.62-1.34)	0.64	No data	
Q		53/78 (68%)	0.80 (0.38–1.66)	0.55	0.73 (0.35-1.55)	0.42	No data	
Interval from malaria to delivery (week)		2505/2987 (84%)	0.97 (0.95–0.99)	0.002	0.98 (0.96-1.00)	0.01	0.96 (0.91-1.01)	0.09
Age (year)		2505/2987 (84%)	0.93 (0.91–0.95)	<0.001	0.91 (0.89-0.94)	<0.001	1.03 (0.97-1.10)	0.35
Parity								
0		1199/1292 (93%)	Reference		Reference		Reference	
1		498/580 (86%)	0.82 (0.58–1.17)	0.27	0.81 (0.56-1.18)	0.27	0.94 (0.30-2.94)	0.92
≥2		807/1112 (73%)	0.59 (0.41–0.86)	0.006	0.65 (0.44-0.96)	0.03	0.43 (0.13-1.43)	0.17
Weight (kg)		2505/2987 (84%)						
Height (cm)		2383/2806 (85%)						
BMI (kg/m^2)		2383/2806 (85%)						
HIV infection	Yes	18/24 (75%)						
	No	2035/2414 (84%)						
Parasitaemia (log_{10}/µL)		2505/2987 (84%)	1.67 (1.42–1.96)	<0.001	1.54 (1.30-1.83)	<0.001	2.39 (1.51-3.77)	<0.001
Body temperature (°C)		2504/2986 (84%)	1.22 (1.00–1.49)	0.045	1.13 (0.90-1.42)	0.28	1.28 (0.84-1.94)	0.25
Haemoglobin (g/dL)		2500/2978 (84%)	0.72 (0.65–0.78)	<0.001	0.70 (0.63-0.77)	<0.001	0.81 (0.62-1.05)	0.12
Gametocytaemia	Yes	84/91 (92%)	3.62 (1.58–8.26)	0.002	4.31 (1.50-12.36)	0.01	3.43 (0.77-15.40)	0.11
	No	2417/2891 (84%)	Reference		Reference		Reference	
Mixed Infection	Yes	12/16 (75%)						
	No	2493/2971 (84%)						
Malaria transmission								
Low		159/249 (64%)	0.81 (0.34–1.91)	0.63	1.20 (0.55-2.58)	0.65		
Moderate		1588/1838 (86%)	Reference		Reference			
High		758/900 (84%)	1.16 (0.63–2.13)	0.64	1.17 (0.67-2.06)	0.58		

AL: artemether-lumefantrine, aOR: adjusted odds ratio, AS: artesunate monotherapy, ASAQ: artesunate-amodiaquine, ASMQ: artesunate-mefloquine, BMI: body mass index, CI: confidence interval, DP: dihydroartemisinin-piperaquine, EGA: estimated gestational age, G: gravidity, HIV: human immunodeficiency virus, OR: odds ratio, Q: quinine monotherapy.
