# Supplementary material for: Pregnancy outcomes and risk of placental malaria after artemisinin-based and quinine-based treatment for uncomplicated falciparum malaria in pregnancy: a WorldWide Antimalarial Resistance Network systematic review and individual patient data meta-analysis
Source: BMC Med. 2020 Jun 2;18:138. doi: 10.1186/s12916-020-01592-z (PMC7263905; doi:10.1186/s12916-020-01592-z)

Additional Figure 3. Forest plot of the proportion of moderate-to-late preterm birth for each study site

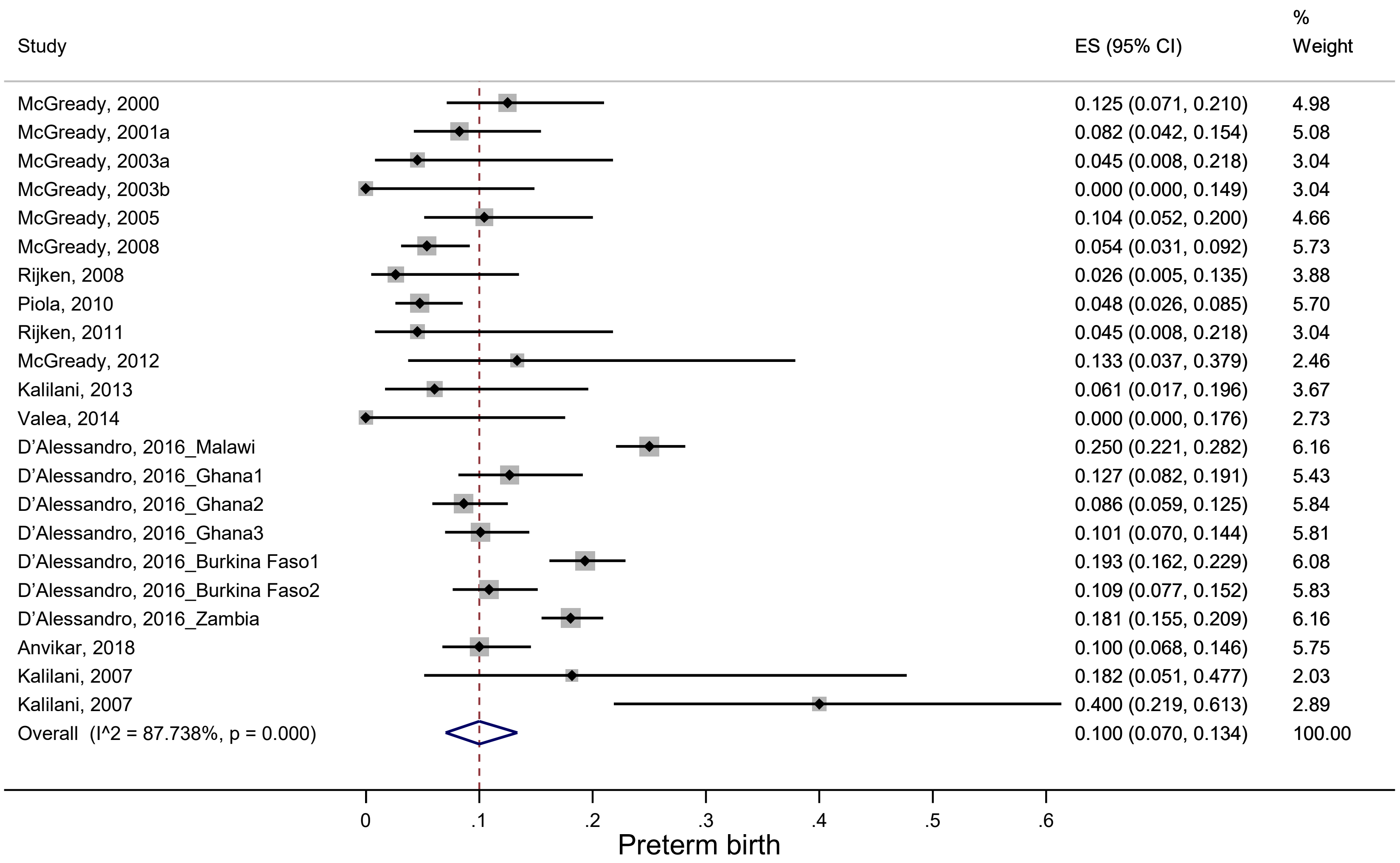

Supplement: Supplementary file 6 — Additional file 6: Additional Figure 3. Forest plot of the proportion of moderate-to-late preterm birth for each study site. [file 12916_2020_1592_MOESM6_ESM.pdf]
