# Supplementary material for: Pregnancy outcomes and risk of placental malaria after artemisinin-based and quinine-based treatment for uncomplicated falciparum malaria in pregnancy: a WorldWide Antimalarial Resistance Network systematic review and individual patient data meta-analysis
Source: BMC Med. 2020 Jun 2;18:138. doi: 10.1186/s12916-020-01592-z (PMC7263905; doi:10.1186/s12916-020-01592-z)

Additional Figure 4. Forest plot of the proportion of small-for-gestational-age for each study site

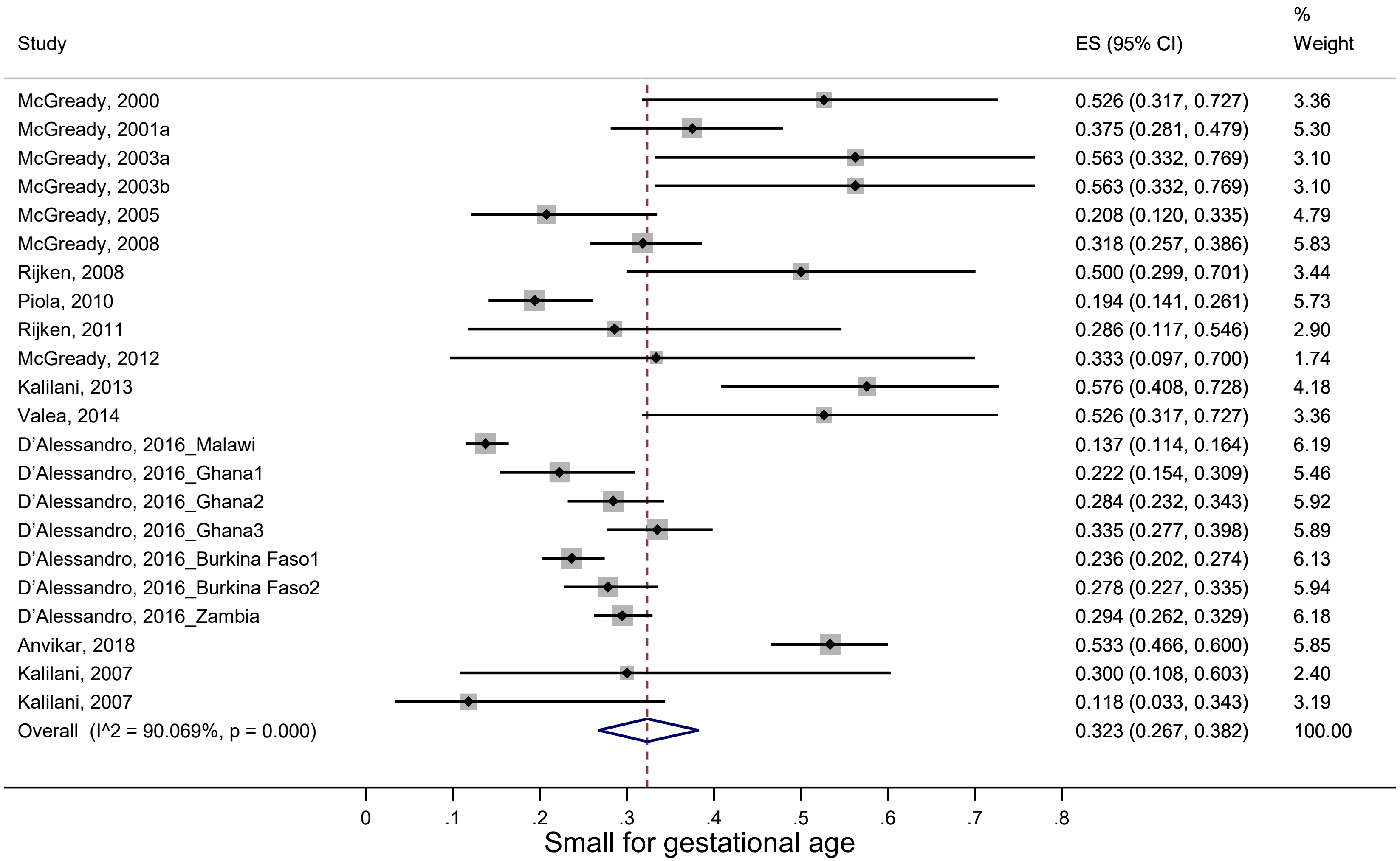

Supplement: Supplementary file 7 — Additional file 7: Additional Figure 4. Forest plot of the proportion of small-for-gestational-age for each study site. [file 12916_2020_1592_MOESM7_ESM.pdf]
