# Supplementary material for: Pregnancy outcomes and risk of placental malaria after artemisinin-based and quinine-based treatment for uncomplicated falciparum malaria in pregnancy: a WorldWide Antimalarial Resistance Network systematic review and individual patient data meta-analysis
Source: BMC Med. 2020 Jun 2;18:138. doi: 10.1186/s12916-020-01592-z (PMC7263905; doi:10.1186/s12916-020-01592-z)
Supplement: Supplementary file 8 — Additional file 8: Additional Table 1. Summary of the studies included in the pooled analyses. [file 12916_2020_1592_MOESM8_ESM.pdf]

Additional Table 1. Summary of the studies included in the pooled analyses

| Study | Study design | Lost-to-follow-up | IPTp | ITN | Gestational age estimation | Outcomes included in the analysis | | | | |
|---|---|---|---|---|---|---|---|---|---|---|
| | | | | | | Congenital abnormality | Miscarriage /stillbirth | Small-for-gestational age | Preterm | Placental histopathology |
| **Shared studies** | | | | | | | | | | |
| McGready, 2000[1] | RCT | 13/111 | Not applicable (Asia) | >90% in this study area* | Fundal height + Dubowitz | Yes | Yes | Yes | Yes | Not assessed |
| McGready, 2001a[2] | RCT | 11/130 | Not applicable (Asia) | >90% in this study area* | Fundal height + Dubowitz | Yes | Yes | Yes | Yes | Not assessed |
| McGready, 2005[3] | RCT | 7/81 | Not applicable (Asia) | >90% in this study area* | Ultrasound | Yes | Yes | Yes | Yes | Yes |
| Kalilani, 2007[4] | RCT | 9/46 | The second dose of the same drug was administered at least 4 weeks after the first dose instead of IPTp | Given to all at enrolment. | Ballard score | Yes | Yes | Yes | Yes | Not assessed |
| McGready, 2008[5] | RCT | 8/246 | Not applicable (Asia) | >90% in this study area* | Ultrasound | Yes | Yes | Yes | Yes | Not assessed |
| Piola, 2010[6] | RCT | 53/292 | Interrupted until delivery | Given to all at enrolment. | Ultrasound | Yes | Yes | Yes | Yes | Yes |
| D'Alessandro, 2016[7, 8] | RCT | 134/3384 | Interrupted for 63 days of follow-up | Given to all at enrolment. | Ultrasound (EGA in week) | Yes | Yes | Yes | Yes | Yes |
| Anvikar, 2018[9] | RCT | 6/244 | Not applicable (Asia) | Information not available | Ultrasound | Yes | Yes | Yes | Yes | Not assessed |
| McGready, 2003a[10] | PK study | 3/27 | Not applicable (Asia) | >90% in this study area* | Fundal height + Dubowitz | Yes | Yes | Yes | Yes | Not assessed |
| McGready, 2012[11] | PK study | 3/19 | Not applicable (Asia) | >90% in this study area* | Ultrasound | Yes | Yes | Yes | Yes | Not assessed |
| Rijken, 2011[12] | PK study | 3/25 | Not applicable (Asia) | >90% in this study area* | Ultrasound | Yes | Yes | Yes | Yes | Not assessed |

Additional Table 1 continued.

| Study | Study design | Lost-to-follow-up | IPTp | ITN | Gestational age estimation | Outcomes included in the analysis | | | | |
|---|---|---|---|---|---|---|---|---|---|---|
| | | | | | | Congenital abnormality | Miscarriage /stillbirth | Small-for-gestational age | Preterm | Placental histopathology |
| Valea, 2014[13] | PK study | 1/21 | IPTp-SP x 2 | Offered to all at enrolment. | LMP or fundal height | Yes | Yes | Yes | Yes | Not assessed |
| Ndiaye, 2011[14] | Single-arm | 0/26 | Information not available | Information not available | Fundal height + Dubowitz (No EGA at delivery) | Yes | No (EGA at delivery is not available) | No (EGA at delivery is not available) | No (EGA at delivery is not available) | Not assessed |
| McGready, 2003b[15] | Observational cohort | 0/24 | Not applicable (Asia) | >90% in this study area* | Fundal height + Dubowitz | Yes | Yes | Yes | Yes | Not assessed |
| Rijken, 2008[16] | Observational cohort | 5/48 | Not applicable (Asia) | >90% in this study area* | Ultrasound | Yes | Yes | Yes | Yes | Not assessed |
| Kalilani, 2013[17, 18] | Observational cohort | 6/41 | IPTp-SP x 3 | Given to all at enrolment. | LMP + fundal height + Ballard score | Yes | Yes | Yes | Yes | Yes |
| **Studies not included** | | | | | | | | | | |
| Adam, 2004a[19] | RCT | 0/42 | Information not available | Information not available | Not specified | Not reported | Assessed | Not assessed | Assessed | Not assessed |
| Adam, 2004c[20] | Single-arm | 0/28 | Information not available | Information not available | Ultrasound | Assessed | Assessed | Not assessed | Assessed | Not assessed |
| Adam, 2006[21] | Single-arm | 0/32 | Information not available | Information not available | Ultrasound | Assessed | Assessed | Not assessed | Assessed | Not assessed |
| Adam, 2012[22] | PK study | 0/12 | Information not available | Information not available | Ultrasound | Assessed | Assessed | Not assessed | Assessed | Not assessed |
| Bounyasong, 2001[23] | RCT | 3/57 | Not applicable (Asia) | Information not available | Ultrasound | Assessed | Not clear | IUGR | EGA at delivery | Calcification of placenta |
| Mutabingwa, 2009[24] | RCT | 5/83 | Information not available | Information not available | Dubowitz | Assessed | Assessed | Not assessed | Assessed | Not assessed |

EGA: estimated gestational age, IPTp: intermittent preventive treatment in pregnancy, ITN: insecticide-treated bed nets, IUGR: intrauterine growth restriction, LMP: last menstrual period, PK: pharmacokinetic, RCT: randomized control trial, SP: sulfadoxine-pyrimethamine
* reported by Boel *et al*. [25]

**Reference for additional files**


1. McGready R, Brockman A, Cho T, Cho D, van Vugt M, Luxemburger C, Chongsuphajaisiddhi T, White NJ, Nosten F: Randomized comparison of mefloquine-artesunate versus quinine in the treatment of multidrug-resistant falciparum malaria in pregnancy. Trans R Soc Trop Med Hyg 2000, 94(6):689-693.
2. McGready R, Cho T, Samuel, Villegas L, Brockman A, van Vugt M, Looareesuwan S, White NJ, Nosten F: Randomized comparison of quinine-clindamycin versus artesunate in the treatment of falciparum malaria in pregnancy. Trans R Soc Trop Med Hyg 2001, 95(6):651-656.
3. McGready R, Ashley EA, Moo E, Cho T, Barends M, Hutagalung R, Looareesuwan S, White NJ, Nosten F: A randomized comparison of artesunate-atovaquone-proguanil versus quinine in treatment for uncomplicated falciparum malaria during pregnancy. J Infect Dis 2005, 192(5):846-853.
4. Kalilani L, Mofolo I, Chaponda M, Rogerson SJ, Alker AP, Kwiek JJ, Meshnick SR: A randomized controlled pilot trial of azithromycin or artesunate added to sulfadoxine-pyrimethamine as treatment for malaria in pregnant women. PLoS One 2007, 2(11):e1166.
5. McGready R, Tan SO, Ashley EA, Pimanpanarak M, Viladpai-Nguen J, Phaiphun L, Wüstefeld K, Barends M, Laochan N, Keereecharoen L *et al*: A randomised controlled trial of artemether-lumefantrine versus artesunate for uncomplicated Plasmodium falciparum treatment in pregnancy. PLoS Med 2008, 5(12):e253.
6. Piola P, Nabasumba C, Turyakira E, Dhorda M, Lindegardh N, Nyehangane D, Snounou G, Ashley Ea, McGready R, Nosten F *et al*: Efficacy and safety of artemether-lumefantrine compared with quinine in pregnant women with uncomplicated Plasmodium falciparum malaria: an open-label, randomised, non-inferiority trial. Lancet Infect Dis 2010, 10(11):762-769.
7. PREGACT Study Group, Pekyi D, Ampromfi AA, Tinto H, Traoré-Coulibaly M, Tahita MC, Valéa I, Mwapasa V, Kalilani-Phiri L, Kalanda G *et al*: Four artemisinin-based treatments in African pregnant women with malaria. N Engl J Med 2016, 374(10):913-927.
8. Nambozi M, Kabuya J-BB, Hachizovu S, Mwakazanga D, Mulenga J, Kasongo W, Buyze J, Mulenga M, Van Geertruyden J-P, D'Alessandro U: Artemisinin-based combination therapy in pregnant women in Zambia: efficacy, safety and risk of recurrent malaria. Malar J 2017, 16:199.
9. Anvikar AR, Kuepfer I, Mishra V, Bruce J, Arya T, Mishra DR, Mohanty S, Mohanty R, Srivastava B, Sharma S *et al*: Efficacy of two artemisinin-based combinations for the treatment of malaria in pregnancy in India: a randomized controlled trial. Malar J 2018, 17:246.
10. McGready R, Stepniewska K, Edstein MD, Cho T, Gilveray G, Looareesuwan S, White NJ, Nosten F: The pharmacokinetics of atovaquone and proguanil in pregnant women with acute falciparum malaria. Eur J Clin Pharmacol 2003, 59(7):545-552.
11. McGready R, Phyo AP, Rijken MJ, Tarning J, Lindegardh N, Hanpithakpon W, Than HH, Hlaing N, Zin NT, Singhasivanon P *et al*: Artesunate/dihydroartemisinin pharmacokinetics in acute falciparum malaria in pregnancy: absorption, bioavailability, disposition and disease effects. Br J Clin Pharmacol 2012, 73(3):467-477.
12. Rijken MJ, McGready R, Phyo AP, Lindegardh N, Tarning J, Laochan N, Than HH, Mu O, Win AK, Singhasivanon P *et al*: Pharmacokinetics of dihydroartemisinin and piperaquine in pregnant and nonpregnant women with uncomplicated falciparum malaria. Antimicrob Agents Chemother 2011, 55(12):5500-5506.
13. Valea I, Tinto H, Traore-Coulibaly M, Toe LC, Lindegardh N, Tarning J, Van Geertruyden J-P, D'Alessandro U, Davies GR, Ward SA: Pharmacokinetics of co-formulated mefloquine and artesunate in pregnant and non-pregnant women with uncomplicated Plasmodium falciparum infection in Burkina Faso. J Antimicrob Chemother 2014, 69(9):2499-2507.
14. Ndiaye JL, Ndiaye A, Faye B, Ba M, Tine R, Ndiaye D, Gaye A, Gaye O, Brasseur P: Open-label in vivo drug study to evaluate the safety and efficacy of artesunate plus amodiaquine

combination in pregnant women with uncomplicated P. falciparum malaria in Senegal. Trop Med Int Health 2011, 16(Supplement 1):140.
15. McGready R, Keo NK, Villegas L, White NJ, Looareesuwan S, Nosten F: Artesunate-atovaquone-proguanil rescue treatment of multidrug-resistant Plasmodium falciparum malaria in pregnancy: a preliminary report. Trans R Soc Trop Med Hyg 2003, 97(5):592-594.
16. Rijken MJ, McGready R, Boel ME, Barends M, Proux S, Pimanpanarak M, Singhasivanon P, Nosten F: Dihydroartemisinin-piperaquine rescue treatment of multidrug-resistant Plasmodium falciparum malaria in pregnancy: a preliminary report. Am J Trop Med Hyg 2008, 78(4):543-545.
17. Cohee LM, Kalilani-Phiri L, Mawindo P, Joshi S, Adams M, Kenefic L, Jacob CG, Taylor TE, Laufer MK: Parasite dynamics in the peripheral blood and the placenta during pregnancy-associated malaria infection. Malar J 2016, 15:483.
18. Kalilani-Phiri L, Thesing PC, Nyirenda OM, Mawindo P, Madanitsa M, Membe G, Wylie B, Masonbrink A, Makwakwa K, Kamiza S *et al*: Timing of malaria infection during pregnancy has characteristic maternal, infant and placental outcomes. PLoS One 2013, 8(9):e74643.
19. Adam I, Ibrahim MH, A/elbasit IA, Elbashir MI: Low-dose quinine for treatment of chloroquine-resistant falciparum malaria in Sudanese pregnant women. East Mediterr Health J 2004, 10(4/5):554-559.
20. Adam I, Elwasila E, Mohammed Ali DA, Elansari E, Elbashir MI: Artemether in the treatment of falciparum malaria during pregnancy in eastern Sudan. Trans R Soc Trop Med Hyg 2004, 98(9):509-513.
21. Adam I, Ali DM, Abdalla MA: Artesunate plus sulfadoxine-pyrimethamine in the treatment of uncomplicated Plasmodium falciparum malaria during pregnancy in eastern Sudan. Trans R Soc Trop Med Hyg 2006, 100(7):632-635.
22. Adam I, Tarning J, Lindegardh N, Mahgoub H, McGready R, Nosten F: Pharmacokinetics of piperaquine in pregnant women in Sudan with uncomplicated Plasmodium falciparum malaria. Am J Trop Med Hyg 2012, 87(1):35-40.
23. Bounyasong S: Randomized trial of artesunate and mefloquine in comparison with quinine sulfate to treat P. falciparum malaria pregnant women. J Med Assoc Thai 2001, 84(9):1289-1299.
24. Mutabingwa TK, Muze K, Ord R, Briceño M, Greenwood BM, Drakeley C, Whitty CJM: Randomized trial of artesunate+amodiaquine, sulfadoxine-pyrimethamine+amodiaquine, chlorproguanal-dapsone and SP for malaria in pregnancy in Tanzania. PLoS One 2009, 4(4):e5138.
25. Boel ME, Rijken MJ, Leenstra T, Phyo AP, Pimanpanarak M, Keereecharoen NL, Proux S, Laochan N, Imwong M, Singhasivanon P *et al*: Malaria in the post-partum period; a prospective cohort study. PLoS One 2013, 8(3):e57890.
26. Adam I, Idris HM, Elbashir MI: Quinine for chloroquine-resistant falciparum malaria in pregnant Sudanese women in the first trimester. East Mediterr Health J 2004, 10(4/5):560-565.
27. Onyamboko MA, Meshnick SR, Fleckenstein L, Koch MA, Atibu J, Lokomba V, Douoguih M, Hemingway-Foday J, Wesche D, Ryder RW *et al*: Pharmacokinetics and pharmacodynamics of artesunate and dihydroartemisinin following oral treatment in pregnant women with asymptomatic Plasmodium falciparum infections in Kinshasa DRC. Malar J 2011, 10:49.
28. Osarfo J, Tagbor H, Cairns M, Alifrangis M, Magnussen P: Dihydroartemisinin-piperaquine versus artesunate-amodiaquine for treatment of malaria infection in pregnancy in Ghana: an open-label, randomised, non-inferiority trial. Trop Med Int Health 2017, 22(8):1043-1052.
