# Supplementary material for: Pregnancy outcomes and risk of placental malaria after artemisinin-based and quinine-based treatment for uncomplicated falciparum malaria in pregnancy: a WorldWide Antimalarial Resistance Network systematic review and individual patient data meta-analysis
Source: BMC Med. 2020 Jun 2;18:138. doi: 10.1186/s12916-020-01592-z (PMC7263905; doi:10.1186/s12916-020-01592-z)
Supplement: Supplementary file 9 — Additional file 9: Additional Table 2. Baseline characteristics of pregnant women assessed for moderate-to-late preterm birth. [file 12916_2020_1592_MOESM9_ESM.pdf]

Additional Table 2. Baseline characteristics of pregnant women assessed for moderate-to-late preterm birth

Characteristic	All		AL		ASAQ		ASMQ		DP	
	N	% (N) Mean (SD)	N	% (N) Mean (SD)	N	% (N) Mean (SD)	N	% (N) Mean (SD)	N	% (N) Mean (SD)
Age group <20	4131	32.0 (1323)	1035	36.2 (375)	747	31.7 (237)	926	28.0 (259)	804	39.8 (320)
20–24		32.2 (1330)		30.7 (318)		31.6 (236)		33.4 (309)		30.1 (242)
25–29		19.4 (803)		18.3 (189)		20.1 (150)		21.5 (199)		16.7 (134)
30–34		9.7 (401)		8.3 (86)		10.8 (81)		10.7 (99)		8.3 (67)
>35		6.6 (274)		6.5 (67)		5.8 (43)		6.5 (60)		5.1 (41)
Gravidity 1	4095	35.1 (1439)	1002	34.6 (347)	746	36.2 (270)	926	32.8 (304)	804	38.4 (309)
2		23.3 (955)		23.0 (230)		22.5 (168)		24.4 (226)		24.4 (196)
≥3		41.5 (1701)		42.4 (425)		41.3 (308)		42.8 (396)		37.2 (299)
Parity 0	4126	42.4 (1750)	1031	41.9 (432)	746	40.8 (304)	926	42.2 (391)	804	47.6 (383)
1		21.7 (896)		20.9 (215)		22.3 (166)		21.0 (194)		22.1 (178)
≥2		35.9 (1480)		37.2 (384)		37.0 (276)		36.8 (341)		30.2 (243)
Height (cm)	3655	155.9 (6.9)	894	156.1 (6.6)	747	158.0 (6.5)	890	156.1 (7.4)	804	155.4 (6.3)
Weight (kg)	4130	53.9 (8.3)	1034	53.7 (7.4)	747	56.3 (8.8)	926	54.0 (8.4)	804	54.5 (8.2)
BMI <18.5	3655	6.1 (224)	894	5.7 (51)	747	5.0 (37)	890	6.4 (57)	804	5.5 (44)
18.5–24.9		80.3 (2936)		85.1 (761)		79.1 (591)		78.3 (697)		78.2 (629)
25.0–29.9		11.8 (431)		8.5 (76)		12.2 (91)		13.5 (120)		14.9 (120)
≥30 (kg/m^2)		1.8 (64)		0.7 (6)		3.7 (28)		1.8 (16)		1.4 (11)
Fever (temperature >37.5°C)	4094	9.2 (375)	1034	8.2 (85)	747	4.6 (34)	925	9.7 (90)	801	4.2 (34)
Haemoglobin on day 0 (g/dL)	4107	10.0 (1.4)	1025	10.2 (1.4)	747	10.0 (1.3)	924	9.9 (1.4)	801	10.0 (1.4)
Parasitaemia (log_{10}/µL)	4131	3.0 (0.9)	1035	3.0 (0.8)	747	2.8 (0.8)	926	3.0 (0.8)	804	2.9 (0.8)
Presence of gametocytes	4075	3.8 (153)	1031	4.1 (42)	747	2.9 (22)	908	1.7 (15)	804	3.7 (30)
Mixed infection	4131	0.7 (29)	1035	0.9 (9)	747	0 (0)	926	0 (0)	804	0.6 (5)
HIV infection	2618	1.2 (31)	771	1.3 (10)	555	0.5 (3)	550	0.2 (1)	642	0.9 (6)
EGA										
<14.0 weeks	4131	0.9 (38)	1035	0.7 (7)	747	0.1 (1)	926	0.4 (4)	804	1.1 (9)
14.0–19.9 weeks		20.9 (863)		24.2 (250)		22.9 (171)		14.4 (133)		21.4 (172)
20.0–23.9 weeks		24.8 (1023)		25.9 (268)		23.3 (174)		24.7 (229)		25.6 (206)
24.0–27.9 weeks		22.2 (917)		20.8 (215)		27.6 (206)		23.9 (221)		19.7 (158)
28.0–36.9 weeks		31.2 (1290)		28.5 (295)		26.1 (195)		36.6 (339)		32.2 (259)

AAP: artesunate with atovaquone-proguanil, AL: artemether-lumefantrine, AS: artesunate monotherapy, ASAQ: artesunate-amodiaquine, ASMQ: artesunate-mefloquine, ASSP: artesunate-sulfadoxine-pyrimethamine, BMI: body mass index, DP: dihydroartemisinin-piperaquine, EGA: estimated gestational age, HIV: human immunodeficiency virus, Q: quinine monotherapy, QC: quinine with clindamycin, SD: standard deviation.

Additional Table 2 continued.

Characteristic	AAP		AS		ASSP		Q		QC	
	N	% (N) Mean (SD)	N	% (N) Mean (SD)	N	% (N) Mean (SD)	N	% (N) Mean (SD)	N	% (N) Mean (SD)
Age group <20	76	28.9 (22)	174	20.1 (35)	147	19.0 (28)	171	21.6 (37)	51	19.6 (10)
20–24		28.9 (22)		23.6 (41)		56.5 (83)		39.2 (67)		23.5 (12)
25–29		21.1 (16)		21.3 (37)		17.0 (25)		20.5 (35)		35.3 (18)
30–34		11.8 (9)		14.4 (25)		4.1 (6)		12.9 (22)		11.8 (6)
>35		9.2 (7)		20.7 (36)		3.4 (5)		5.8 (10)		9.8 (5)
Gravidity 1	76	31.6 (24)	174	27.0 (47)	147	47.6 (70)	169	32.5 (55)	51	25.5 (13)
2		19.7 (15)		15.5 (27)		25.2 (37)		25.4 (43)		25.5 (13)
≥3		48.7 (37)		57.5 (100)		27.2 (40)		42.0 (71)		49.0 (25)
Parity 0	76	35.5 (27)	174	29.9 (52)	147	55.8 (82)	171	36.3 (62)	51	33.3 (17)
1		23.7 (18)		19.5 (34)		23.1 (34)		25.7 (44)		25.5 (13)
≥2		40.8 (31)		50.6 (88)		21.1 (31)		38.0 (65)		41.2 (21)
Height (cm)	32	149.9 (8.2)	128	151.6 (5.7)	116	150.0 (7)	44	153.3 (5.1)	0	
Weight (kg)	76	49.7 (6.6)	174	48.9 (5.9)	147	47.7 (6.9)	171	55.1 (9.4)	51	49.4 (5.4)
BMI <18.5	32	3.1 (1)	128	7.8 (10)	116	18.1 (21)	44	6.8 (3)	0	
18.5–24.9		81.3 (26)		84.4 (108)		72.4 (84)		90.9 (40)		
25.0–29.9		9.4 (3)		7.0 (9)		9.5 (11)		2.3 (1)		
≥30 (kg/m^2)		6.3 (2)		0.8 (1)		0 (0)		0 (0)		
Fever (temperature >37.5°C)	76	25.0 (19)	174	27.0 (47)	115	24.3 (28)	171	15.8 (27)	51	21.6 (11)
Haemoglobin on day 0 (g/dL)	75	9.3 (1.5)	174	9.5 (1.5)	145	9.2 (1.4)	166	10.2 (1.8)	50	9.2 (1.7)
Parasitaemia (log_{10}/µL)	76	3.6 (1)	174	3.4 (1)	147	3.4 (0.8)	171	3.4 (0.9)	51	3.3 (1.1)
Presence of gametocytes	76	3.9 (3)	171	10.5 (18)	116	3.4 (4)	171	8.2 (14)	51	9.8 (5)
Mixed infection	76	0 (0)	174	6.9 (12)	147	0 (0)	171	0 (0)	51	5.9 (3)
HIV infection	0		0		23	17.4 (4)	77	9.1 (7)	0	
EGA										
<14.0 weeks	76	2.6 (2)	174	4.6 (8)	147	2.0 (3)	171	2.3 (4)	51	0 (0)
14.0–19.9 weeks		22.4 (17)		20.1 (35)		18.4 (27)		28.1 (48)		19.6 (10)
20.0–23.9 weeks		19.7 (15)		18.4 (32)		25.9 (38)		30.4 (52)		17.6 (9)
24.0–27.9 weeks		18.4 (14)		14.9 (26)		23.1 (34)		17.0 (29)		27.5 (14)
28.0–36.9 weeks		36.8 (28)		42.0 (73)		30.6 (45)		22.2 (38)		35.3 (18)

AAP: artesunate with atovaquone-proguanil, AL: artemether-lumefantrine, AS: artesunate monotherapy, ASAQ: artesunate-amodiaquine, ASMQ: artesunate-mefloquine, ASSP: artesunate-sulfadoxine-pyrimethamine, BMI: body mass index, DP: dihydroartemisinin-piperaquine, EGA: estimated gestational age, HIV: human immunodeficiency virus, Q: quinine monotherapy, QC: quinine with clindamycin, SD: standard deviation.
