# Supplementary material for: Pregnancy outcomes and risk of placental malaria after artemisinin-based and quinine-based treatment for uncomplicated falciparum malaria in pregnancy: a WorldWide Antimalarial Resistance Network systematic review and individual patient data meta-analysis
Source: BMC Med. 2020 Jun 2;18:138. doi: 10.1186/s12916-020-01592-z (PMC7263905; doi:10.1186/s12916-020-01592-z)
Supplement: Supplementary file 10 — Additional file 10: Additional Table 3. Baseline characteristics of pregnant women assessed for small-for-gestational-age. [file 12916_2020_1592_MOESM10_ESM.pdf]

Additional Table 3. Baseline characteristics of pregnant women assessed for small-for-gestational-age

Characteristic	All		AL		ASAQ		ASMQ		DP	
	N	% (N)<br>Mean (SD)	N	% (N)<br>Mean (SD)	N	% (N)<br>Mean (SD)	N	% (N)<br>Mean (SD)	N	% (N)<br>Mean (SD)
Age group <20	3707	32.4 (1202)	973	37.5 (365)	700	31.9 (223)	820	27.7 (227)	716	40.6 (291)
20–24		32.0 (1185)		29.8 (290)		31.0 (217)		33.7 (276)		30.0 (215)
25–29		19.3 (717)		17.9 (174)		20.1 (141)		21.3 (175)		16.1 (115)
30–34		9.6 (357)		8.3 (81)		11.0 (77)		10.5 (86)		8.2 (59)
>35		6.6 (246)		6.5 (63)		6.0 (42)		6.8 (56)		5.0 (36)
Gravidity 1	3671	35.8 (1316)	940	35.5 (334)	699	36.2 (253)	820	33.5 (275)	716	40.1 (287)
2		22.9 (839)		23.2 (218)		22.0 (154)		23.7 (194)		23.5 (168)
≥3		41.3 (1516)		41.3 (388)		41.8 (292)		42.8 (351)		36.5 (261)
Parity 0	3702	43.1 (1596)	969	43.1 (418)	699	40.8 (285)	820	42.6 (349)	716	49.0 (351)
1		21.1 (780)		20.4 (198)		21.7 (152)		20.6 (169)		21.2 (152)
≥2		35.8 (1326)		36.4 (353)		37.5 (262)		36.8 (302)		29.7 (213)
Height (cm)	3347	156.0 (6.9)	855	156.1 (6.5)	700	158.0 (6.6)	813	156.2 (7.4)	716	155.4 (6.2)
Weight (kg)	3707	54.1 (8.2)	973	53.7 (7.2)	700	56.1 (8.7)	820	54.1 (8.4)	716	54.7 (8.2)
BMI <18.5	3347	6.0 (201)	855	4.9 (42)	700	5.1 (36)	813	7.0 (57)	716	5.2 (37)
18.5–24.9		80.3 (2687)		85.5 (731)		79.4 (556)		78.4 (637)		77.8 (557)
25.0–29.9		12.0 (400)		8.9 (76)		11.7 (82)		12.9 (105)		15.6 (112)
≥30 (kg/m^2)		1.8 (59)		0.7 (6)		3.7 (26)		1.7 (14)		1.4 (10)
Fever (temperature >37.5°C)	3677	8.8 (325)	973	7.8 (76)	700	5.0 (35)	819	9.4 (77)	715	4.3 (31)
Haemoglobin on day 0 (g/dL)	3690	10.0 (1.4)	964	10.2 (1.3)	700	10.0 (1.3)	820	9.9 (1.3)	714	10.0 (1.4)
Parasitaemia ($\log_{10}$/µL)	3707	3.0 (0.8)	973	3.0 (0.8)	700	2.8 (0.8)	820	3.0 (0.8)	716	2.9 (0.8)
Presence of gametocytes	3655	3.6 (133)	970	4.0 (39)	700	3.1 (22)	801	1.2 (10)	716	3.6 (26)
Mixed infection	3707	0.7 (25)	973	1.0 (10)	700	0 (0)	820	0 (0)	716	0.1 (1)
HIV infection	2437	1.0 (24)	726	1.1 (8)	521	0.6 (3)	514	0.2 (1)	595	1.0 (6)
EGA										
<14.0 weeks	3707	0.8 (31)	973	0.7 (7)	700	0 (0)	820	0.2 (2)	716	1.0 (7)
14.0–19.9 weeks		20.6 (765)		23.6 (230)		23.3 (163)		13.0 (107)		21.2 (152)
20.0–23.9 weeks		24.7 (914)		25.7 (250)		23.4 (164)		25.5 (209)		25.6 (183)
24.0–27.9 weeks		22.1 (820)		21.2 (206)		27.6 (193)		24.1 (198)		19.3 (138)
28.0–36.9 weeks		30.7 (1138)		27.4 (267)		25.7 (180)		36.7 (301)		33.0 (236)
≥37.0 weeks		1.1 (39)		1.3 (13)		0 (0)		0.4 (3)		0 (0)

AAP: artesunate with atovaquone-proguanil, AL: artemether-lumefantrine, AS: artesunate monotherapy, ASAQ: artesunate-amodiaquine, ASMQ: artesunate-mefloquine, ASSP: artesunate-sulfadoxine-pyrimethamine, BMI: body mass index, DP: dihydroartemisinin-piperaquine, EGA: estimated gestational age, HIV: human immunodeficiency virus, Q: quinine monotherapy, QC: quinine with clindamycin, SD: standard deviation.

Additional Table 3 continued.

Characteristic	AAP		AS		ASSP		Q		QC	
	N	% (N)<br>Mean (SD)	N	% (N)<br>Mean (SD)	N	% (N)<br>Mean (SD)	N	% (N)<br>Mean (SD)	N	% (N)<br>Mean (SD)
Age group <20	55	27.3 (15)	154	16.2 (25)	129	20.2 (26)	119	20.2 (24)	41	14.6 (6)
20–24		25.5 (14)		27.9 (43)		55.8 (72)		40.3 (48)		24.4 (10)
25–29		25.5 (14)		23.4 (36)		17.8 (23)		21.0 (25)		34.1 (14)
30–34		12.7 (7)		13.6 (21)		3.9 (5)		11.8 (14)		17.1 (7)
>35		9.1 (5)		18.8 (29)		2.3 (3)		6.7 (8)		9.8 (4)
Gravidity 1	55	29.1 (16)	154	25.3 (39)	129	50.4 (65)	117	31.6 (37)	41	24.4 (10)
2		20.0 (11)		15.6 (24)		24.8 (32)		23.9 (28)		24.4 (10)
≥3		50.9 (28)		59.1 (91)		24.8 (32)		44.4 (52)		51.2 (21)
Parity 0	55	32.7 (18)	154	27.9 (43)	129	58.9 (76)	119	37.0 (44)	41	29.3 (12)
1		21.8 (12)		20.1 (31)		22.5 (29)		22.7 (27)		24.4 (10)
≥2		45.5 (25)		51.9 (80)		18.6 (24)		40.3 (48)		46.3 (19)
Height (cm)	23	148.0 (8.5)	107	151.7 (5.6)	102	150.0 (7.2)	31	153.4 (5.4)	0	
Weight (kg)	55	50.9 (7.3)	154	50.0 (6.6)	129	47.8 (7.1)	119	55.6 (9.3)	41	49.3 (5.5)
BMI <18.5	23	78.3 (0)	107	7.5 (8)	102	18.6 (19)	31	6.5 (2)	0	
18.5–24.9		13.0 (18)		83.2 (89)		70.6 (72)		87.1 (27)		
25.0–29.9		8.7 (3)		8.4 (9)		10.8 (11)		6.5 (2)		
≥30 (kg/m^2)		0 (2)		0.9 (1)		0 (0)		0 (0)		
Fever (temperature >37.5°C)	55	25.5 (14)	154	25.3 (39)	101	25.7 (26)	119	15.1 (18)	41	22.0 (9)
Haemoglobin on day 0 (g/dL)	54	9.2 (1.4)	154	9.5 (1.5)	127	9.2 (1.3)	116	10.4 (1.7)	41	9.3 (1.7)
Parasitaemia (log_{10}/µL)	55	3.6 (1.1)	154	3.3 (1.1)	129	3.4 (0.8)	119	3.4 (0.8)	41	3.3 (1.1)
Presence of gametocytes	55	5.5 (3)	151	11.3 (17)	102	2.9 (3)	119	8.4 (10)	41	7.3 (3)
Mixed infection	55	1.8 (1)	154	7.1 (11)	129	0 (0)	119	0 (0)	41	4.9 (2)
HIV infection	0		0		21	14.3 (3)	60	5.0 (3)	0	
EGA										
<14.0 weeks	55	3.6 (2)	154	4.5 (7)	129	1.6 (2)	119	3.4 (4)	41	0 (0)
14.0–19.9 weeks		21.8 (12)		22.1 (34)		19.4 (25)		29.4 (35)		17.1 (7)
20.0–23.9 weeks		18.2 (10)		16.2 (25)		26.4 (34)		28.6 (34)		12.2 (5)
24.0–27.9 weeks		18.2 (10)		10.4 (16)		20.9 (27)		18.5 (22)		24.4 (10)
28.0–36.9 weeks		30.9 (17)		38.3 (59)		31.8 (41)		18.5 (22)		36.6 (15)
≥37.0 weeks		7.3 (4)		8.4 (13)		0 (0)		1.7 (2)		9.8 (4)

AAP: artesunate with atovaquone-proguanil, AL: artemether-lumefantrine, AS: artesunate monotherapy, ASAQ: artesunate-amodiaquine, ASMQ: artesunate-mefloquine, ASSP: artesunate-sulfadoxine-pyrimethamine, BMI: body mass index, DP: dihydroartemisinin-piperaquine, EGA: estimated gestational age, HIV: human immunodeficiency virus, Q: quinine monotherapy, QC: quinine with clindamycin, SD: standard deviation.
