# Supplementary material for: Pregnancy outcomes and risk of placental malaria after artemisinin-based and quinine-based treatment for uncomplicated falciparum malaria in pregnancy: a WorldWide Antimalarial Resistance Network systematic review and individual patient data meta-analysis
Source: BMC Med. 2020 Jun 2;18:138. doi: 10.1186/s12916-020-01592-z (PMC7263905; doi:10.1186/s12916-020-01592-z)
Supplement: Supplementary file 11 — Additional file 11: Additional Table 4. Baseline characteristics of pregnant women assessed for deposition of malaria pigment in the placenta. [file 12916_2020_1592_MOESM11_ESM.pdf]

Additional Table 4. Baseline characteristics of pregnant women assessed for deposition of malaria pigment in the placenta

Characteristic	All		AL		AS		ASAQ		ASMQ		DP		Q	
	N	% (N)<br>Mean (SD)	N	% (N)<br>Mean (SD)	N	% (N)<br>Mean (SD)	N	% (N)<br>Mean (SD)	N	% (N)<br>Mean (SD)	N	% (N)<br>Mean (SD)	N	% (N)<br>Mean (SD)
Interval from malaria to delivery (week)	3033	14.1 (6.1)	893	14.3 (6.2)	85	13.8 (8.0)	649	14.7 (5.8)	668	13.5 (5.7)	658	14.0 (6.2)	80	14.6 (6.7)
Age group <20	3033	34.7 (1051)	893	37.6 (336)	85	15.3 (13)	649	31.1 (202)	668	31.9 (213)	658	40.4 (266)	80	26.3 (21)
20–24		30.3 (920)		29.1 (260)		22.4 (19)		31.7 (206)		30.1 (201)		30.4 (200)		42.5 (34)
25–29		18.7 (567)		18.4 (164)		18.8 (16)		20.0 (130)		19.3 (129)		16.7 (110)		22.5 (18)
30–34		9.8 (296)		9.0 (80)		18.8 (16)		10.6 (69)		11.1 (74)		8.1 (53)		5.0 (4)
>35		6.6 (199)		5.9 (53)		24.7 (21)		6.5 (42)		7.6 (51)		4.4 (29)		3.8 (3)
Parity 0	3030	43.3 (1311)	891	43.5 (388)	85	24.7 (21)	648	40.9 (265)	668	42.1 (281)	658	49.1 (323)	80	41.3 (33)
1		19.6 (595)		19.0 (169)		17.6 (15)		20.5 (133)		17.7 (118)		21.1 (139)		26.3 (21)
≥2		37.1 (1124)		37.5 (334)		57.6 (49)		38.6 (250)		40.3 (269)		29.8 (196)		32.5 (26)
Height (cm)	2845	156.6 (6.7)	785	156.2 (6.6)	85	151.6 (5.7)	649	158.0 (6.6)	668	157.2 (7.2)	658	155.7 (6.2)	0	
BMI (kg/m^2)	2845	22.3 (3.0)	785	21.7 (2.4)	85	21.6 (2.5)	649	22.5 (3.3)	668	22.5 (3.2)	658	22.7 (2.9)	0	
Body temperature (°C)	3032	36.7 (0.6)	893	36.7 (0.6)	85	37.0 (1.1)	649	36.7 (0.6)	667	36.7 (0.5)	658	36.5 (0.5)	80	37.1 (0.6)
Haemoglobin on day 0 (g/dL)	3024	10.1 (1.4)	885	10.2 (1.4)	85	9.6 (1.5)	649	10.1 (1.3)	668	10.1 (1.3)	658	10.1 (1.3)	79	10.5 (1.6)
Parasitaemia (log_{10}/µL)	3033	3.0 (0.8)	893	3.1 (0.8)	85	3.5 (1.0)	649	2.8 (0.8)	668	2.9 (0.8)	658	2.9 (0.8)	80	3.3 (0.9)
Presence of gametocytes	3028	3.0 (91)	890	3.1 (28)	83	8.4 (7)	649	3.2 (21)	668	0.9 (6)	658	3.0 (20)	80	11.3 (9)
Mixed infection	3033	0.5 (16)	893	0.8 (7)	85	10.6 (9)	649	0.0 (0)	668	0.0 (0)	658	0.0 (0)	80	0.0 (0)
HIV infection	2315	1.1 (25)	700	1.4 (10)	0		479	0.6 (3)	497	0.0 (0)	577	1.2 (7)	62	8.1 (5)
Treatment outcome														
No recurrence	2987	74.6 (2227)	880	60.1 (529)	84	82.1 (69)	645	81.2 (524)	658	72.3 (476)	642	87.7 (563)	78	84.6 (66)
Recrudescence		3.3 (98)		5.6 (49)		8.3 (7)		1.7 (11)		3.6 (24)		0.9 (6)		1.3 (1)
Reinfection		20.0 (596)		32.2 (283)		8.3 (7)		15.2 (98)		21.6 (142)		9.7 (62)		5.1 (4)
No PCR		2.2 (66)		2.2 (19)		1.2 (1)		1.9 (12)		2.4 (16)		1.7 (11)		9.0 (7)
MAP category														
Low	3033	8.3 (252)	893	13.5 (121)	85	100.0 (85)	649	0.0 (0)	668	0.0 (0)	658	0.0 (0)	80	57.5 (46)
Moderate		61.9 (1877)		58.0 (518)		0.0 (0)		54.1 (351)		54.5 (364)		92.7 (610)		42.5 (34)
High		29.8 (904)		28.4 (254)		0.0 (0)		45.9 (298)		45.5 (304)		7.3 (48)		0.0 (0)

AL: artemether-lumefantrine, AS: artesunate monotherapy, ASAQ: artesunate-amodiaquine, ASMQ: artesunate-mefloquine, BMI: body mass index, DP: dihydroartemisinin-piperaquine, HIV: human immunodeficiency virus, Q: quinine monotherapy, SD: standard deviation.
