# Supplementary material for: Pregnancy outcomes and risk of placental malaria after artemisinin-based and quinine-based treatment for uncomplicated falciparum malaria in pregnancy: a WorldWide Antimalarial Resistance Network systematic review and individual patient data meta-analysis
Source: BMC Med. 2020 Jun 2;18:138. doi: 10.1186/s12916-020-01592-z (PMC7263905; doi:10.1186/s12916-020-01592-z)
Supplement: Supplementary file 12 — Additional file 12: Additional Table 5. Multivariable logistic regression on the risk of deposition of malaria pigment in the placenta by parity in different malaria transmission area. [file 12916_2020_1592_MOESM12_ESM.pdf]

Additional Table 5. Multivariable logistic regression on the risk of deposition of malaria pigment in the placenta by parity in different malaria transmission area

	aOR (95% CI)*	p-value
Low transmission		
Parity 0	Reference	
1	1.22 (0.53–2.82)	0.64
≥2	1.26 (0.62–2.59)	0.53
Moderate transmission		
Parity 0	Reference	
1	0.71 (0.46–1.11)	0.13
≥2	0.52 (0.34–0.80)	0.003
High transmission		
Parity 0	Reference	
1	0.84 (0.39–1.79)	0.65
≥2	0.50 (0.26–0.98)	0.04

CI: confidence interval, aOR: adjusted odds ratio.

* Adjusted for treatment outcome, interval from malaria to delivery, age, body temperature, haemoglobin on day 0, parasitaemia and presence of gametocyte. p-value for interaction: 0.20.
